# SeDeM as a Tool to Validate Drug Substance Manufacturing Processes and Assess Scalability and Suitability for Direct Compression: Supplier Screening

**DOI:** 10.3390/pharmaceutics15082034

**Published:** 2023-07-28

**Authors:** Alba Figuera-Figuera, Marc Suñé-Pou, Pilar Pérez-Lozano, Encarna García-Montoya, Joaquim Amela-Navarro, Josep M. Suñé-Negre

**Affiliations:** 1 Pharmaceutical Technology and Physico-Chemical Department, Universitat de Barcelona, Av. Joan XXIII, 27-31, 08028 Barcelona, Spain; albafiguera099@gmail.com (A.F.-F.); perezlo@ub.edu (P.P.-L.); encarnagarcia@ub.edu (E.G.-M.); jamelan@ub.edu (J.A.-N.); jmsune@ub.edu (J.M.S.-N.); 2 IDIBELL-UB Research Group, Pharmacotherapy, Pharmacogenomics and Pharmaceutical Technology, Avinguda Granvia, 199-203, 08908 L’Hospitalet de Llobregat, Spain

**Keywords:** drug substance manufacturers, particle size, SeDeM expert system, critical quality attribute, critical material attribute, direct compression, preformulation, Linezolid, powder characterization, process validation

## Abstract

During the development of an oral solid form of a drug substance, a thorough understanding of the critical material attributes is necessary, as the physical properties of the active pharmaceutical ingredient (API) can profoundly influence the drug product’s manufacturability, critical quality attributes, and bioavailability. The objective of this study was to validate the manufacturing process of the drug Linezolid from three different sources at both the pilot and industrial scale and to identify differences in critical material attributes between the API manufacturers. Furthermore, the scalability factor between the pilot and industrial scale and the suitability of a process for direct compression were also evaluated. In the present study, the different sources of API were characterized by SeDeM methodology, particle size distribution, and scanning electron microscopy determinations. The statistical analysis revealed that no statistically significant differences were found for any of the parameters under study for the same API source analyzed on both scales. On the other hand, for most of the parameters evaluated, statistical differences were observed between the different sources. It was concluded that SeDeM was able to successfully validate the API manufacturing process, assess scalability, and distinguish between sources. Therefore, it could be highly valuable in the formulation phase to select the best API source.

## 1. Introduction

The successful implementation of a quality-by-design (QbD) approach for safe, effective, and quality pharmaceutical products requires a deeper scientific understanding of the materials, products, and processes [1,2]. As the ICH Q8 (R2) and ICH Q11 guidelines suggest, in addition to the characterization of critical process parameters (CPPs), a systematic physicochemical characterization of the materials involved in the process and the critical material attributes (CMAs) for drug substances and drug products should also be performed. The purpose is to identify and understand the key aspects that influence the quality of the drug substances and drug products. By combining these two parameters, CPPs and CMAs, a design space can be estimated to meet the specifications given by the critical quality attributes (CQAs) derived from the quality target product profile (QTPP) [3,4].

The production of active pharmaceutical ingredients (APIs) is usually through a synthetic organic chemistry procedure that should be safe, robust, and cost-effective. The manufacture of small-molecule APIs often requires the production of multiple intermediates using different complex processes, which implies a tedious and costly operation [5,6,7].

Once a robust, reproducible, and optimized synthesis method is achieved, the aim is to transfer the drug substance to the industrial scale and validate the process at that scale. It is widely recognized that the scale-up process is time-consuming and technically complex [8].

One of the most striking aspects of the validation and manufacturing of a drug substance is the fact that the critical quality attributes of the drug substance are usually established based on chemical characteristics such as its assay, purity, elemental impurities, chiral purity, related substances, and residual solvents [9,10]. In contrast, physical properties such as bulk and tapped densities, particle shape and size, particle size distribution, flowability, compressibility, and cohesivity attract relatively little attention and are not examined in detail [6,11,12,13]. This entails the risk that industrial batches of the API may not have the desired repetitive physical and galenic characteristics, leading to a different performance during the manufacturing process. It could, therefore, compromise the processability and the critical quality attributes of the drug product. This has a huge impact on the cost and production time of medicines in the pharmaceutical industry [14,15,16,17].

In order to design and develop a robust drug product that has the intended CQAs, the physical, chemical, and biological properties of the drug substance must be carefully considered. Mechanical and physical properties, although often not studied in detail during the development of drug substances and in the pre-formulation steps, can have a profound impact on the formulation development and processing of solid dosage forms [18,19]. A good early understanding of the critical material mechanical properties and variability of the drug substance allows for a rational, risk-based selection of the drug product formulation and manufacturing process. In addition, it can be useful in developing a more efficient and cheaper processing method, such as direct compression or roller compaction manufacturing, instead of wet granulation [2,20]. It also permits the rational selection of the most appropriate excipients based on drug substance properties, and helps to assess the critical material attributes and root cause analysis during scale-up or process failure. An understanding of these factors should help reduce development times, complexity, and risk in late-stage development and full-scale manufacturing [15].

In view of the above, we raise the question of whether a suitable tool with simple determinations could ensure the reproducibility at the pilot and industrial scale in the production of a drug substance. Furthermore, this tool should complement the chemical characterization performed by the manufacturer and establish the critical attributes of the materials for the manufacturing of the drug substance. In this paper, the SeDeM expert system was used with the aim to address the above-mentioned requirements.

The SeDeM diagram expert system is a pre-formulation system applied for the development of solid dosage forms, which characterizes powdered substances (API, excipients, and intermediates, such as granules, pellets, bulk blend, and mixtures) on the basis of various parameters related to the critical material attributes (flowability, compressibility, lubricity, and particle distribution), which have an impact on the critical quality attributes of the drug product and its quality [21,22,23,24,25,26,27,28,29]. It is widely used as a tool to assess the suitability of powder substances to be processed by direct compression (DC), and to identify the properties of the materials to be improved for their use in direct compression. The SeDeM system also allows, by means of various mathematical equations, the calculation of the quantity of excipients with certain characteristics required for the correction of a particular property in order to obtain a final blend suitable for direct compression [30,31,32,33,34,35,36,37,38,39,40]. Moreover, it has also proven to be very useful for comparing the reproducibility among batches of the same material through the following system indices: parametric index, parametric profile index, and good compressibility index (PI, PPI, and GCI, respectively) [21].

The SeDeM expert system may be considered as both time- and cost-saving, as this technique may reduce the number of trials and optimize the development time.

The objective of this study was to apply the SeDeM expert system to validate the drug substance manufacturing process from different sources at the pilot and industrial scale and to identify potential differences between various API manufacturers. Moreover, the scalability factor between the pilot and industrial scale was assessed, in addition to the suitability of the process for direct compression.

The drug substance under study was Linezolid, which was chosen as a drug model to be analyzed here and to validate the model. Linezolid is a synthetic hospital antibiotic approved by the FDA for the treatment of nosocomial and community-acquired severe infections caused by Gram-positive bacteria resistant to other antibiotics (Streptococci, Vancomycin-resistant enterococci (VRE), and methicillin-resistant Staphylococcus aureus (MRSA)) [41,42,43]. The recommended dose is one film-coated tablet (600 mg Linezolid) twice daily; tablets should be swallowed whole with water, as directed by a patient leaflet [44]. In view of the above, it is important to note that Linezolid tablets contain a high drug substance load per tablet, and, in order to facilitate their intake and swallowing by patients, the weight of the tablet should not be increased significantly. Considering the strength of Linezolid, the drug substance’s physical properties and critical material attributes will have a profound impact on the formulation development, manufacturability, and processing of the tablets, as well as the tablets’ CQA. In addition, to facilitate drug product intake, the quantity of excipients should be minimized. The SeDeM expert system was used in this work to establish the suitability of this drug substance to be processed by DC. DC is the tablet production process of choice because of its simplified and shorter steps compared to other technologies such as wet or dry granulation, resulting in cost savings, improved stability, and reduced production costs.

## 2. Materials and Methods

### 2.1. Materials

The following materials were used in this study: Linezolid from three different manufacturers: Glenmarck Pharmaceuticals Pvt. Ltd., Mumbai, India; USV Pvt. Ltd., Mumbai, India; and UQUIFA—Unión Químico Farmacéutica S.A, Barcelona, Spain. Two different batch sizes from each source were evaluated, one at pilot scale (batch size of approximately 2 kg) and one at industrial scale (batch size of approximately 25 kg). Details of the batches studied from each source are shown below:

- Glenmark: GL-1, GL-2, GL-3 (pilot scale), GL-4, GL-5, and GL-6 (industrial scale);
- USV: US-1, USV-2, US-3 (pilot), US-4, US-5, and US-6 (industrial scale);
- UQUIFA: UQ-1, UQ-2, UQ-3 (pilot scale), UQ-4, UQ-5, and UQ-6 (industrial scale).

### 2.2. Methods

In the first set of experiments, pilot-scale material was used, and the research was focused on establishing and understanding the differences between all sources from a particle and pharmacotechnical characterization perspective. Furthermore, the objective was to verify the reproducibility of the synthetic route of obtention of each of the manufacturers investigated, and to validate the manufacturing process at the pilot scale. The SeDeM expert system methodology was employed with the evaluation of the twelve critical material attributes, together with particle size distribution (PSD) and morphology analysis (SEM) determinations.

Once the experiments at pilot scale were completed, the next step was to check whether the manufacturing process for all sources of API examined at the industrial scale was also robust and reproducible at this scale. Additionally, the aim of this step was to establish the equivalence between the pilot scale and industrial scale and to confirm the scale transposition of the method of obtention of all sources investigated. In this set of experiments, SeDeM was used to assess the reproducibility of all sources and to confirm the scale transposition.

It is worth mentioning that, as the SeDeM expert system is a powerful tool to establish the suitability of powders (API and excipients) to be processed with direct compression technology, the suitability of the different sources of drug substances for DC was also assessed.

#### 2.2.1. API Characterization on the Basis of SeDeM Expert System

The drug substances from the three manufacturers were characterized in terms of the different parameter tests required by the SeDeM expert system. Whenever possible, the compendial methods reported in the pharmacopeias were applied. Some of the parameters were determined experimentally according to the established procedure and some were calculated on the basis of other basic parameters as per Table 1 [21].

The mean value of each parameter test was obtained and used in the radius calculations as described below. A SeDeM diagram was drawn for each source and selected excipient. (All analyses were performed in triplicate and the values represent an average measurement.

The parameters considered in the SeDeM method are as follows:

- Bulk density (Da): This parameter was determined according to method described in section 2.9.34 of the European Pharmacopeia using a settling apparatus with a graduated cylinder (voluminometer).
- Tapped density (Dc): This parameter was determined according to method described in section 2.9.34 of the European Pharmacopeia using a settling apparatus with a graduated cylinder (voluminometer). The volume taken was the value obtained after 2500 strokes.
- Inter-particle porosity (Ie): It is calculated from the following equation: Ie = Dc − Da/Dc × Da, described in Table 1 [21].
- Carr index (IC): It is calculated from the following equation: IC = (Dc − Da/Dc) × 100, described in Table 1 [21].
- Cohesion index (Icd): This index was experimentally determined by directly compressing the powder under study, preferably in an eccentric press. The hardness (N) was determined, and the mean hardness values calculated. Initially, the powder is tested by itself, but if it is abrasive (it cannot be compressed), 3.5% of the following standard lubrication should be added to the powder: talc 2.36%, Aerosil^®^ 200 0.14%, and magnesium stearate 1.00% [21].
- Hausner ratio (IH): This method is described in section 2.9.34 of the European Pharmacopeia. This is calculated from Da and Dc as: IH = Dc/Da.
- Angle of repose (α): The method is described in section 2.9.36 of the European Pharmacopeia. This is the angle of the cone formed when the product is passed through a funnel with the following dimensions: height 9.5 cm, upper diameter of spout 7.2 cm, internal diameter at the bottom, and narrow end of spout 1.8 cm. The funnel is placed on a support 20 cm above the table surface, centered over a millimeter-grid sheet on which two intersecting lines are drawn, crossing at the center. The spout is plugged and the funnel is filled with the product until it is flush with the top end of the spout when smoothed with a spatula. Remove the plug and allow the powder to fall onto the millimeter sheet. Measure the four radii of the cone base with a slide caliper and calculate the mean value (r). Measure the cone height formed (h). Deduce α from tan (α) = h/r.
- Flowability (t″): This parameter was in accordance with the one described in section 2.9.16 of the European Pharmacopeia. It is expressed in seconds and tenths of a second per 100 g of sample, with the mean value of three measurements.
- Loss on drying (%HR): This parameter was measured using the method described in section 2.2.32 in the European Pharmacopeia. The samples (1 g) were dried in an oven at 105 °C ± 2 °C, until a constant weight was obtained.
- Hygroscopicity (%H): It is the determination of the percentage increase in sample weight (1 g powder) after being kept in a humidifier at a relative humidity of 76% (±2%) and a temperature of 22 °C ± 2 °C for 24 h.
- Percentage of particles measuring < 50 μm (%Pf): Particle size was determined by means of the sieve test following the general method 2.9.12 of the European Pharmacopeia. The value returned is the % of particles that pass through a 0.05 mm sieve when vibrated for 10 min at speed 10 (CISA^®^ vibrator).
- Homogeneity index (Iθ): This was calculated according to the General method 2.9.12 of the European Pharmacopeia. To determine particle size by means of the sieve test, the grain size of a 100 g sample was measured by subjecting a sieve stack to vibration for 10 min at speed 10 (CISA^®^ vibrator). The sieve sizes used are 0.355 mm, 0.212 mm, 0.100 mm, and 0.05 mm. The percentage of product retained in each sieve is calculated and the amount that passes through the 0.05 mm sieve is measured. The percentage of fine particles (<50 μm) (%Pf) was calculated as described above. Note that if this percentage is higher than that calculated in the complete sieve test, it is because some of the particles adhered/stuck to the product retained in the sieves during the grain-size test, and the percentage of <50 μm particles found may be lower than the true figure. The following equation is then applied to the data obtained (Equation (1) named in Table 1).

Equation (1), mentioned in Table 1, is as follows:

(1) $$I\theta =\frac{\mathrm{Fm}}{100+\left(\mathrm{dm}\;-\;\mathrm{dm}-1\right)\mathrm{Fm}-1+\left(\mathrm{dm}+1-\;\mathrm{dm}\right)\mathrm{Fm}+1+\left(\mathrm{dm}\;-\;\mathrm{dm}-2\right)\mathrm{Fm}-2+\left(\mathrm{dm}+2-\;\mathrm{dm}\right)\mathrm{Fm}-2+\ldots +\left(\mathrm{dm}\;-\;\mathrm{dm}-n\right)\mathrm{Fm}-n\;+\left(\mathrm{dm}+n\;-\;\mathrm{dm}\right)\mathrm{Fm}+n}$$

where Iθ is the relative homogeneity index, representing the particle-size homogeneity in the range of the fractions under study:

Fm: Percentage of particles in the majority range;
Fm − 1: Percentage of particles in the range immediately below the majority range;
Fm + 1: Percentage of particles in the range immediately above the majority range;
n: Order number of the fraction under study, within a series, with respect to the majority fraction;
dm: Mean diameter of the particles in the majority fraction;
dm−1: Mean diameter of the particles in the fraction of the range immediately below the majority range;
dm + 1: Mean diameter of the particles in the fraction of the range immediately above the majority range.

The methods used for each test are extensively described in the literature [21,22,23,24]:

Once the numerical values of the different parameters were obtained following the specific methods, the numeric values for each SeDeM diagram parameter were converted into a radius value (r), based on known equations of the SeDeM method, as given in Table 1 [26].

The SeDeM diagram was constructed on the basis of 12 parameters in the form of a 12-sided polygon. When all radius values were 10, the SeDeM diagram took the form of a circumscribed regular polygon, drawn by connecting the radius values with linear segments. The results obtained from the earlier parameter calculations and conversions, graphically represented by the radius, were used to draw the SeDeM diagrams. A diagram was formed by connecting radius values with linear segments, taking 0 as the minimum value, 10 as the maximum value, and 5 as the minimum acceptable value [24].

To determine whether or not the product is acceptable for direct compression in numerical form, the following indices were calculated based on the SeDeM diagram: parameter index (IP), parameter profile index (IPP), and good compression index (GCI).

-  Parameter index (IP) = No × p ≥ 5/No × Pt
  No × p ≥ 5: Indicates the number of parameters whose value is equal to or higher than 5.
  No × Pt: Indicates the total number of parameters studied.
  The acceptability limit would correspond to IP ≥ 5.
-  Parameter profile index (IPP) = mean r ≥ 5 of all parameters
  Mean r = mean value of the parameters calculated.
  The acceptability limit would correspond to IPP = mean r ≥ 5.
-  Good compression index (GCI) = IPP × f
  where f is the reliability factor and is calculated as follows:
  f = polygon area/circle area
  The acceptability limit was calculated using GCI = IPP × f > 5.

#### 2.2.2. Particle Size and Particle Size Distribution by Laser Diffraction

The determination of the particle size and particle size distribution of the drug substances was performed following the general method given in the 2.9.31 European Pharmacopeia. The test was carried out by means of a laser diffraction analyzer (Mastersizer 2000, Malvern, UK) fitted with a dry sampler unit (SIROCCO 2000SM, Malvern, UK). Data were evaluated using Mastersizer 2000 v5.60 software (Malvern, UK). The following conditions were used:

Material:
  - Material name: Linezolid
  - Refractive index: 1.590
Measurement:
  - Measurement time: 12 s
  - Measurement snaps: 12,000
  - Background time: 12 s
  - Background snaps: 12,000
Sampler settings:
  - Sampler tray: General Purpose
  - Dispersion feed ratio:
    -  Vibration feed Rate: 70%
    -  Dispersive air Pressure: 3.125
Measurement cycles:
  - Aliquots: 1 per SOP:1
  - Measurement: 1 per aliquot

Three readings were performed for each measure.

#### 2.2.3. Determination of Particle Morphology by Scanning Electron Microscopy (SEM)

The morphology (shape and surface) of the investigated drug substances from different manufacturers was examined using scanning electron microscopy (SEM) (Hitachi S-4100 FE-SEM, Hitachi High-Technologies Europe, Krefeld, Germany) operating at an accelerating voltage of 15 kV.

#### 2.2.4. Statistical Analysis

A statistical study was conducted to analyze and compare the intra- and inter-batch variability in the radius of all of the critical material attributes investigated with the SeDeM method, for all sources. STATGRAPHICS Centurion XVI (Statgraphics Technologies, Inc., The Plains, VA, USA) was used for the data evaluation, with the one-way ANOVA analysis test.

## 3. Results and Discussion

### 3.1. Pilot-Scale Batches

#### 3.1.1. Drug Substance Characterization on the Basis of SeDeM Expert System

The radius and corresponding incidence factors (individual values and mean) obtained according to the described methodology for each critical material attribute of Glenmarck, USV, and UQUIFA studied at the pilot scale are summarized in Table 2, Table 3 and Table 4. Each parameter was determined in triplicate and converted into the radius, and the mean value of the radius was used in the comparison between the different batches from the same source.

**Figure 1:**
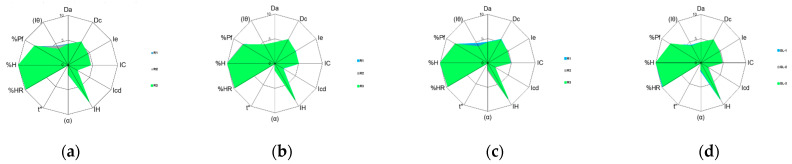
SeDeM diagram of Linezolid Glenmarck batches: (**a**) the shaded area corresponds to superimposition of R1, R2, and R3 of batch GL-1; (**b**) the shaded area corresponds to superimposition of R1, R2, and R3 of batch GL-2; (**c**) the shaded area corresponds to superimposition of R1, R2, and R3 of batch GL-3; and (**d**) the shaded area corresponds to the superimposition of mean values of GL-1, GL-2, and GL-3.

**Figure 2:**
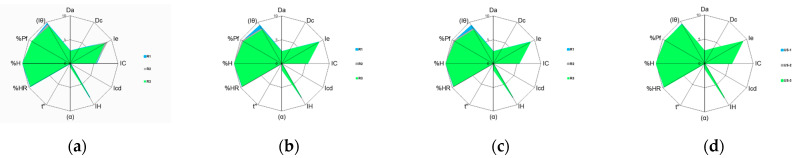
SeDeM diagram of Linezolid USV batches: (**a**) the shaded area corresponds to the superimposition of R1, R2, and R3 of batch US-1; (**b**) the shaded area corresponds to the superimposition of R1, R2, and R3 of batch US-2; (**c**) the shaded area corresponds to the superimposition of R1, R2, and R3 of batch US-3; and (**d**) the shaded area corresponds to the superimposition of mean values of US-1, US-2, and US-3.

**Figure 3:**
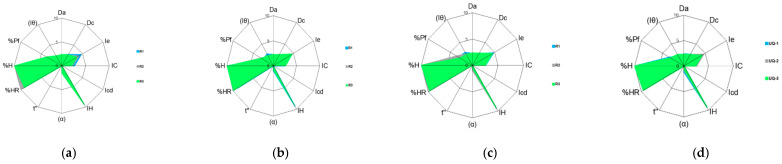
SeDeM diagram of Linezolid UQUIFA batches: (**a**) the shaded area corresponds to the superimposition of R1, R2, and R3 of batch UQ-1; (**b**) the shaded area corresponds to the superimposition of R1, R2, and R3 of batch UQ-2; (**c**) the shaded area corresponds to the superimposition of R1, R2, and R3 of batch UQ-3; and (**d**) the shaded area corresponds to the superimposition of mean values of UQ-1, UQ-2, and UQ-3.

**Figure 4:**
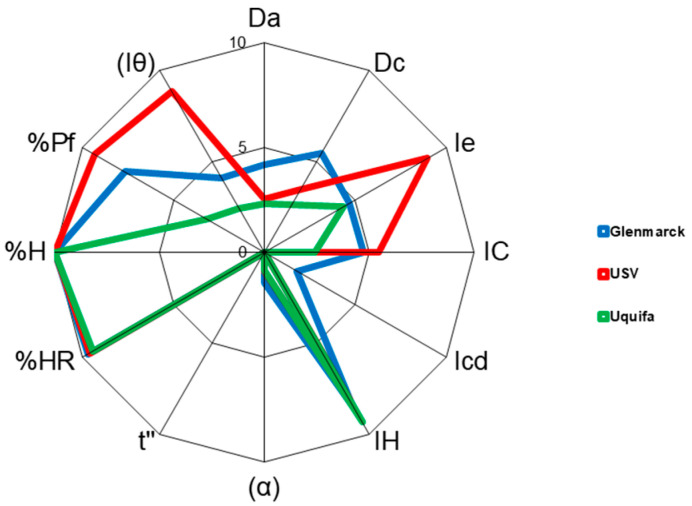
SeDeM diagram superimposition of mean radius (R) values of the different sources pilot scale batches: Glenmarck (blue), USV (red), and UQUIFA (green).

Additionally, the SeDeM diagrams of each manufacturer with the individual radius obtained (three replicates) for each of the three batches and the mean radius are also depicted in Figure 1, Figure 2 and Figure 3, respectively. A comparison of the SeDeM profile diagram between all sources at the pilot scale can be seen in Figure 4.

The test results obtained were statistically processed using the Statgraphics Centurion XVI Software Package, in order to verify the possible equivalence between the three batches of drug substance studied for each manufacturer and to establish the differences among sources. Table 5 shows the mean ($\bar{x}$), the variance (S2), the standard deviation (Sn − 1), and the coefficient of variation (CV%) for each parameter and for the PP and IGC indices, calculated based on the standard deviation for n-1 elements. Moreover, different statistical analyses were performed to determine the kind of statistics (parametric or non-parametric) to be applied, for which Levene’s test was used. For all sources and for all parameters, it was demonstrated that parametric statistics can be used for all the critical material attributes under study. The analysis of variance was first verified with Levene’s tests (Table 6), and a one-way analysis of variance (ANOVA) was used for the statistical study of all the parameters and indices.

As is detailed in Table 5, low variability was found between the three batches of each drug substance source for all the critical material attributes, which implies that the nine values were consistent with each other and showed low dispersion. Therefore, this confirms that, with the SeDeM method, it would be possible to extrapolate information based on a few batches to the whole population. Consequently, with simple determinations, it would be possible to predict the behavior of the drug substance, and, since all the sources showed high repeatability, it would not be necessary to perform a full analysis when it comes to critical material attributes. The angle of repose was found to be the parameter that showed the highest variability, and the worst result was found with UQUIFA. This could be attributed to the poor flowability of Linezolid for all the manufacturers (all the sources showed an angle of repose of more than 40°), and the smaller particle size and narrower particle size distribution of UQUIFA compared to other sources [45].

As shown in Table 6, no statistically significant difference (*p* > 0.05) was found for any of the parameters under study of each of the drug substance sources tested. Therefore, all critical parameters were found to be reproducible within the same source. It can be concluded that the reproducibility of the method of obtention was confirmed for each of the manufacturers examined, and the variation between batches within the same source can be considered minimal.

On top of the statistical treatment, as can be seen in Figure 1, Figure 2, Figure 3 and Figure 4, each manufacturer showed a unique diagram that clearly differs from the other sources. Taken together with the radius (Table 2, Table 3 and Table 4), these results indicate that, although all the drug substances were chemically identical, they exhibited different physical properties, which can lead to differences in the way they will affect the processing of the drug product. These differences can be attributed to the use of different synthetic routes, small variations in the purification or crystallization steps, and different solvents [5,6,7]. Likewise, it is also well-described in the literature that post-crystallization unit operations, which occur at the end of the manufacturing process (filtration, drying, milling, and further procedures), can cause changes in the physical properties [11,46,47,48]. The differences between manufacturers will be discussed further in Section 3.4.

This study demonstrated that the SeDeM expert system can be used to distinguish between the different manufacturers of API, and that it is useful tool to validate the process on a small scale of production. As described by Pérez et al. [21], it is reinforced that it can be used to validate the manufacturing process of drug substances.

#### 3.1.2. Particle Size Distribution (PSD) Results

**Figure 5:**
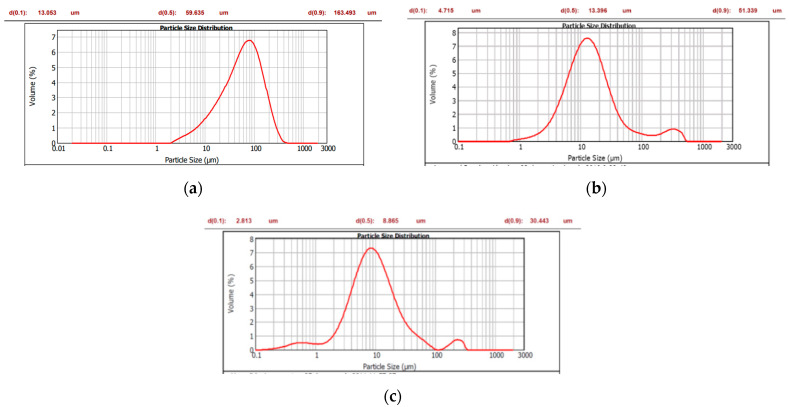
(**a**) Particle size distribution of Linezolid Glenmarck (Batch GL-2); (**b**) particle size distribution of Linezolid USV (Batch US-2); (**c**) particle size distribution of Linezolid UQUIFA (Batch UQ-2).

It is a well-known fact that particle size and particle size distribution, along with the particle morphology of API, have a huge impact on the processability of the drug substance during the drug product manufacturing process. As illustrated in Figure 5a, Glenmarck not only had substantially larger particles than those obtained for USV and UQUIFA, but also had a larger distribution as can be seen in Table 7 (F’ of 12.525) compared to the other sources, ranging from 13 to 163.5 µm. The distribution is bell-shaped and symmetrical, and presents a slight bias to the left.

As for the USV source, Figure 5b and Table 7 reveal a similar particle size to UQUIFA, slightly larger and with a wider distribution curve (F’ of 10.888) from 4.7 to 51.4 µm. The UQUIFA source showed the smallest particle size and the narrowest particle size distribution, as can be seen in Figure 5c and Table 7.

#### 3.1.3. SEM Results

**Figure 6:**
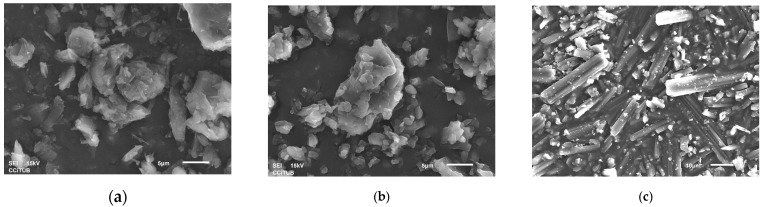
(**a**) SEM images of Linezolid Glenmarck (Batch GL-2); (**b**) SEM images of Linezolid USV (Batch US-2); (**c**) SEM images of UQUIFA (Batch UQ-2).

The surface morphology of the particles of different API sources was studied using the scanning electron microscopy technique (SEM). The SEM micrograph of the Glenmarck source (Figure 6a) showed larger particles with a platelet-like shape and some needle-like crystals on the surface. UQUIFA exhibited an acicular or needle-like shape (Figure 6c). USV depicted a prismatic platelet-like shape closer to spheres compared to the other sources (Figure 6b) and exhibited surface roughness.

### 3.2. Industrial Scale

#### Drug Substance Characterization on the basis of the SeDeM Expert System

One of the questions to be answered in this work was whether the results obtained at the pilot scale could be confirmed at the industrial scale. As noted in the introduction to this paper, it is widely known that the scaling-up of the manufacturing process for a drug substance can be challenging, as the synthetic chemical route involves several steps [7,8]. In addition, dissimilar times are required to complete the process, and, together with the different equipment used, this could compromise the achievement of a product with the desired critical quality attributes [11].

**Figure 7:**
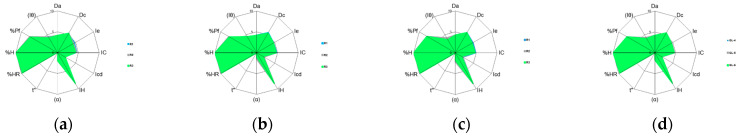
SeDeM diagram of Linezolid Glenmarck batches: (**a**) the shaded area corresponds to the superimposition of R1, R2, and R3 of batch GL-4; (**b**) the shaded area corresponds to the superimposition of R1, R2, and R3 of batch GL-5; (**c**) the shaded area corresponds to the superimposition of R1, R2, and R3 of batch GL-6; and (**d**) the shaded area corresponds to the superimposition of mean values of GL-4, GL-5, and GL-6.

**Figure 8:**
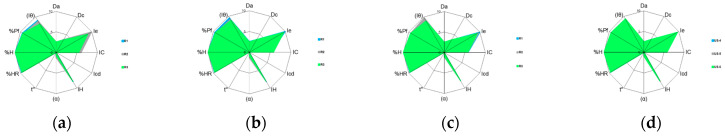
SeDeM diagram of Linezolid USV batches: (**a**) the shaded area corresponds to the superimposition of R1, R2, and R3 of batch US-4; (**b**) the shaded area corresponds to the superimposition of R1, R2, and R3 of batch US-5; (**c**) the shaded area corresponds to the superimposition of R1, R2, and R3 of batch US-6; and (**d**) the shaded area corresponds to the superimposition of mean values of US-4, US-5, and US-6.

**Figure 9:**
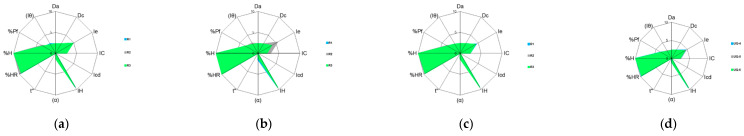
SeDeM diagram of Linezolid UQUIFA batches: (**a**) the shaded area corresponds to the superimposition of R1, R2, and R3 of batch UQ-4; (**b**) the shaded area corresponds to the superimposition of R1, R2, and R3 of batch UQ-5; (**c**) the shaded area corresponds to the superimposition of R1, R2, and R3 of batch UQ-6; and (**d**) the shaded area corresponds to the superimposition of mean values of UQ-4, UQ-5, and UQ-6.

**Figure 10:**
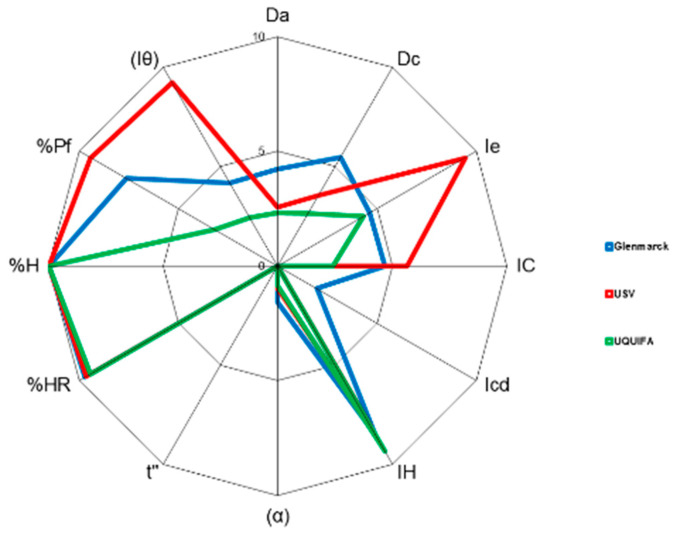
SeDeM diagram superimposition of mean radius (R) values of the different sources industrial scale batches: Glenmarck (blue), USV (red), and UQUIFA (green).

Therefore, in order to corroborate the previous pilot results, the different API sources at the industrial scale (25 kg) were characterized following the SeDeM methodology described in Section 2.2.1. The radius and incidence factors of the three sources studied at the industrial scale are summarized in Table 8, Table 9 and Table 10. Each parameter was determined in triplicate (three times) and converted into the radius, and the mean value of the radius was used in the comparison between the different batches of the same source. The SeDeM diagrams of each manufacturer with individual radius obtained (three replicates) for each of the three batches and mean radius are also depicted in Figure 7, Figure 8 and Figure 9, respectively. A comparison of the SeDeM profile diagram between all sources at the industrial scale can be seen in Figure 10.

The test results were statistically treated using the Statgraphics Centurion XVI Software Package, in order to assess the equivalence between the three batches of drug substance studied for each manufacturer, and to establish the differences among sources at the industrial scale. In Table 11, the mean ($\bar{x}$), variance (S2), standard deviation of the mean (Sn − 1), and coefficient of variation (CV%) for each parameter and for the PP and IGC indices are shown, calculated based on the standard deviation for n − 1 elements. In addition, different statistical analyses were performed to determine the kind of statistics (parametric or non-parametric) to be applied, for which Levene’s test was used. It was demonstrated that parametric statistics can be used for all of the critical material attributes and all sources under study. The analysis of variance was first checked with Levene’s tests (Table 12), and a one-way analysis of variance (ANOVA) was used for the statistical study of all the parameters and indices.

As illustrated in Table 11, in terms of the radius of the different industrial sources, slightly better results were obtained for the twelve critical material attributes of all analyzed sources compared to the pilot-scale ones. It was noteworthy that there was less inter- and intra-batch variability, and it was reiterated that the angle of repose parameter showed the widest variation. Nonetheless, at the industrial scale, all sources examined exhibited a similar spreading of the results for this parameter. One explanation for this might be that, due to the shape and size of the particles, the drug substance has poor flow properties, and showed more variability in this parameter [14,49,50,51].

As can be seen from Table 12, no statistically significant differences (*p* > 0.05) were found between the three batches of each API source studied. The results found with the three batches of the same API demonstrate that each of the sources, Glenmarck, USV, and UQUIFA, were able to consistently produce sufficiently reliable and reproducible values. It was verified that all of the manufacturers produced a drug substance of the intended quality that met the material critical attribute requirements (ICHQ7) [52]. In addition, batch-to-batch reproducibility, drug substance quality, and the robustness and reproducibility of production methods were ensured. Therefore, it can be concluded that SeDeM can also be used to validate the manufacturing process at an industrial scale.

As regards the SeDeM diagrams depicted in Figure 7, Figure 8, Figure 9 and Figure 10, each drug substance source displayed a unique and characteristic profile (like the source’s fingerprint) drawn by the variations in the different material attributes examined. All of this demonstrates that the results obtained at the pilot scale were corroborated at an industrial scale for all tested sources of API. The differences observed in the critical material attributes studied proved that each of the sources exhibited different physical properties, which may lead to a different impact on the processability of the drug product [53,54]. Although all sources are chemically identical and complied with the chemical test specifications described in the certificate of analysis, they showed dissimilar physical behavior that may affect the manufacturing process and critical quality attributes of the drug product [15,55]. Therefore, the drug substance’s sourcing should be closely monitored.

### 3.3. Scale Transposition

The third objective of this research was to establish the equivalence between the pilot and industrial scale, and to confirm the scale transposition of the process from a critical material attributes point of view. Having demonstrated that the SeDeM system can be used to validate the manufacturing process of drug substances from different sources at the pilot and industrial scale, the objective was to verify the reproducibility of results between small and large batch sizes. For this purpose, the mean values of the three pilot batches of each source were compared with those of the three industrial scale batches (Table 13 and Table 14).

As the development of an API moves from pilot-scale to large-scale manufacturing, it becomes more challenging to maintain the physicochemical properties [13]. There are several factors that can contribute to this, such as changes in the batch size and equipment during scale-up, resulting in changes in processing parameters. There may also be changes in the synthetic route to improve batch purity and yield, as drug product development progresses through the different stages [50].

According to the results obtained in Table 14, no significant statistical differences were observed between the six batches tested (three at the pilot scale and three at the industrial scale) from each source and for all the parameters assessed. Therefore, the correct scale transposition between the pilot and industrial scale was confirmed for all the sources examined. For all sources of the drug substance tested, regardless of source or scale, reproducibility at the pilot and industrial scale and transposition between the small and large scale was demonstrated for all the critical material attributes related to the physical drug substance properties. The dimensions, compressibility, flowability, lubrication/stability, and lubrication/dosage incidence factors showed similar results. The PP and IGC presented consistent results during the scale-up step, with slightly better results even being obtained at the industrial scale.

It can be concluded that SeDeM, aside from ensuring the reproducibility of the manufacturing process, has proven to be a useful tool to verify the scalability of the manufacturing process, and to distinguish between different sources of the same drug substance.

### 3.4. Suitability of API for the Direct Compression and Analysis of Critical Material Attributes

The Table 15 shows a comparison of all critical material quality attributes at pilot and industrial scale as well as the incidence factors for all the suppliers. In addition, parameter index (IP), parameter profile index (IPP), and good compression index (GCI) are also presented.

**Figure 11:**
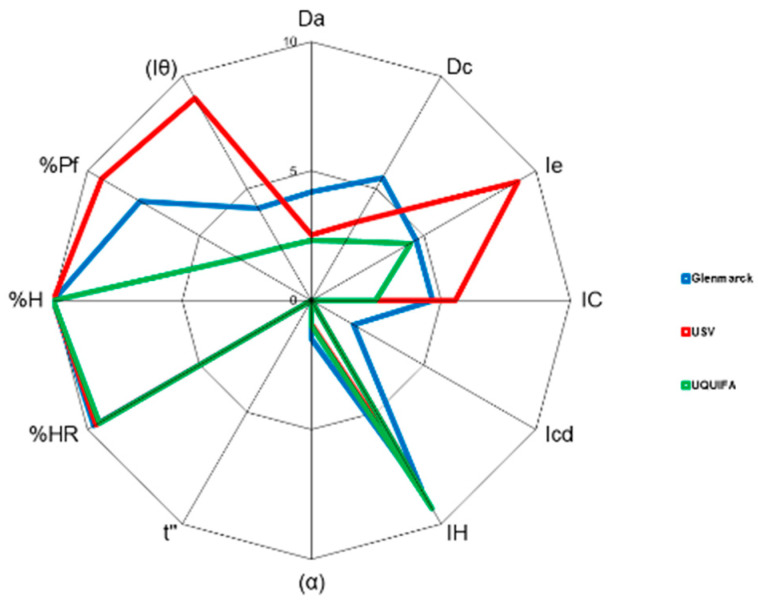
SeDeM diagram superimposition of mean radius (R) values of the different sources (pilot and industrial batches): Glenmarck (blue), USV (red), and UQUIFA (green).

As can be seen in Table 16 and Figure 11, for most of the parameters analyzed, statistical differences were observed between the different sources (*p* < 0,005). The only parameter that seemed to be equivalent for all sources was the %HR. These results could be attributed to the differences observed in the PSD and SEM tests, the dissimilar particle shape detected (presence of platelet, needle-like, acicular, or prismatic particles), particle size and particle size distribution, and surface variations.

It is well-reported that properties such as particle size and shape can have an impact on the critical material attributes of dimensions (Da, Dc), compressibility (Ie, IC, Icd), flowability (IH, α, t″), and lubricity/dosage (%Pf, Iθ) [56,57]. The small particle size and needle-like morphology can lead to issues such as poor flowability [58], difficulties in blending [57], and undesirable adhesion to surfaces such as tablet punches [59].

Popov et al. [60] examined the effect of particle shape on flowability and found that, as a particle becomes more needle-like, or the aspect ratio (defined as particle length to particle width) increases, so does the resistance to flow, which could lead to differences in the pile up, cohesiveness, and compressibility.

Podczeck and Miah [61] reported that flowability depends on the particle geometry, with flowability increasing as the shape changes from needle-like to cubic, to angular, and to round particles, due to the interparticle attraction. Particle size also has an effect on flowability. Tan and Newton [62] reported that flowability generally decreases with decreasing particle size.

In view of the above, it is important to study the critical material attributes in the early stages of development and to assess the differences in these CMAs between different sources before introducing a new source or switching from one to another. The perceived significant differences between sources may lead to different behavior of the drug substances at different steps of the drug product manufacturing process. This could involve the use of different equipment [63], variations or deviations from the registered and validated manufacturing process, and the adjustment of the critical process parameters so as not to affect the CQA. As an example, differences in densities and flowability can be a contributing factor to product processability, particularly in the compression of powdered material. This may result in issues in the filling of the die, or segregation in the feeder during compression, among other problems. This, in turn, could lead to issues in the uniformity of the weight and content of the tablets, defects in the tablets, and erratic values of hardness, disintegration, and dissolution. Therefore, it would be necessary to adjust the critical process parameters of the tabletting process so as not to affect the critical attributes of the tablets.

A more detailed analysis of the industrial-scale results in Table 15, and the SeDeM diagram study for the different API sources at the industrial scale (Figure 10 and Table 11) indicate that it is a substance with poor flowability/rheological properties (mean incidence radius 3.35, 3.00, and 3.45), limited compressibility (mean incidence radius 3.75, 5.02, and 2.34), and dimensions (4.84, 3.06, and 2.46). As regards lubrication and stability, this was the only incidence factor in which all sources exhibited an excellent radius for loss in the drying and hygroscopicity parameters; all of the values were above 9.70 as a mean value. As for the dosage/lubrication incidence, dissimilar results were obtained for the three sources. USV exhibited excellent values of 9.43 (particles < 50 µm) and 9.24 (homogeneity index), which demonstrate the excellent suitability of this source for direct compression. At the other extreme was UQUIFA, with values below 5 for both parameters: 3.39 (particles < 50 µm) and 2.32 (homogeneity index). This could be attributed to the large population of particles below 50 µm (Table 7). Concerning Glenmarck, as can be seen in Table 15, it showed an acceptable result for % Pf (7.63) but a radius below 5 for the homogeneity index; this means that the percentage of particles below 50 µm was adequate but the distribution between different sieve sizes was not the most desirable. This reinforces the results obtained in the PSD test.

The SeDeM expert system, in combination with the PSD test and SEM analysis, proved to be a useful preformulation tool to distinguish between different API sources. It is worth mentioning that, although all API sources meet the particle size distribution specifications, some differences in their behavior were detected during the processing of the drug substance and mixtures. This could be attributed to the fact particle shape (larger platelet needle-like crystal prismatic), interparticle interactions, roughness, and its density and porosity also have a significant impact, and this could potentially cause issues during the tabletting step and affect drug product critical quality attributes.

From what has been presented in this section, considering also the results obtained at the pilot and industrial scale for all sources studied, it is evident that the USV source was the most suitable to be processed using direct compression, and, thus, it was selected for further studies. Consequently, in order to formulate a suitable blend for direct compression with this USV source, an excipient must be used which, when used in the smallest possible quantity, improves the poor SeDeM indices and parameters, and, particularly, the flowability, dimensions, and compressibility parameters.

The poor flowability of Linezolid can be improved by the addition of a suitable diluent that has a good flow property and can contribute to the flowability of the powder mixer. Alternatively, the addition of a glidant will improve the flowability of different sources of Linezolid. However, poor compactibility is a significant challenge in the case of Linezolid. For the successful formulation of Linezolid tablets, it is important to prepare a powder mixture that has sufficient mechanical strength to withstand post-compressional stress during coating, packaging, and shipment. This can be achieved by the addition of a directly compressible diluent possessing good compaction properties. Considering the high drug substance loading per tablet, the quantity of excipient should be kept to a minimum in order not to significantly increase the tablet weight. Therefore, appropriate excipients should be selected that, with the minimum quantity, confer the desired properties to the API mixture to compensate for the dimensions in which Linezolid API has shortcomings.

As regards the production of tablets by direct compression and the quantity of excipients required to produce them, it was only possible to manufacture tablets with the desired properties that met the drug product critical quality attributes with the USV source. The suitability of this source for DC processing was confirmed and reinforces the results obtained in the SeDeM expert diagram at the pilot and industrial scale (data available in Tables S19 and S20 of the Supplementary Material).

For Glenmarck and UQUIFA, it was not possible to manufacture the tablets, as a huge quantity of corrective excipients was required to compensate for the low incidence factors. Tablets produced with Glenmarck failed the resistance to crushing and friability tests, as they showed low hardness, and, with the UQUIFA source, it was not technically feasible to produce any tablet. Considering the fact that Linezolid tablets have a high drug loading content, it would be challenging to improve the above-mentioned issues with a reasonable quantity of excipients. Therefore, these two sources were considered unsuitable for DC technology.

## 4. Conclusions

The present study demonstrated that the SeDeM expert system is a suitable tool to verify the reproducibility of the manufacturing process for the Linezolid drug substance at the pilot and industrial scale, and validated the manufacturing process. The results obtained also indicate that it can be used to assess the scalability between manufacturing processes for drug substances. In addition, it was validated as a useful tool for the characterization of powdered substances on the basis of their critical physical parameters, and it was able to distinguish between sources. The system was successfully applied to determine the profile of the drug model Linezolid from different sources and to establish its suitability for direct compression. The differences in particle size and shape properties may have a significant impact on the unit operations during the manufacturing process (cohesiveness and compressibility), as well as the critical quality attributes and the stability of the drug product. Based on the above, SeDeM could be helpful in assessing and predicting whether these changes in critical material attributes could affect the direct compression manufacturing process. Thus, it has been proven as a powerful tool for contributing to the formulation of dosage forms and the proper selection of the manufacturing method.

All Linezolid sources (Glenmarck, USV, and UQUIFA) demonstrated a high reproducibility of results obtained at the pilot and industrial scale between manufacturers. Furthermore, the scalability between two batch sizes was confirmed for all of them. Nevertheless, statistically significant differences were found between manufacturers. Based on the results of the SeDeM characterization, Glenmarck and UQUIFA were found to not be suitable for DC. The USV source showed the best results for IGC and the greatest suitability for DC.

## Figures and Tables

**Table 1:** Parameters and equations used in the SeDeM expert system, conversion limits, and applied factors.

Incidence	Parameter	Symbol	Unit	Equation	Limit Value	Radius (r)	Applied Factor to v
Dimensions	Bulk density	Da	g/mL	Da = P/Va	0–1 g/mL	0–10	10 v
	Tapped density	Dc	g/mL	Dc = P/Vc	0–1 g/mL	0–10	10 v
Compressibility	Interparticle porosity	Ie	(-)	Ie = Dc − Da/Dc × Da	0–1.2	0–10	10 v/1.2
	Carr index	IC	%	IC = (Dc − Da/Dc) 100	0–50 (%)	0–10	v/5
	Cohesion index ^a^	Icd	N	Experimental	0–200 (N)	0–10	v/290
Flowability	Hausner ratio	IH	(-)	IH = Dc/Da	1–3	10–0	5 (3 − v)
	Angle of repose	(α)	º	tg α = h/r	50–0 (º)	0–10	10 − (v/5)
	Flowability	t″	s	Experimental	20–0 (s)	0–10	10 − (v/2)
Lubrication/stability	Loss on drying	%HR	%	Experimental	0–10 (%)	10–0	10 − v
	Hygroscopicity	%H	%	Experimental	20–0 (%)	0–10	10 − (v/2)
Dosage/lubrication	Particles < 50 µm	%Pf	µ	Experimental	50–0 (%)	0–10	10 − (v/5)
	Homogeneity index ^b^	(Iθ)	-	(Equation (1))	0–2 ×10^−2^	0–10	500 v

^a^ Hardness (N) of the tablets obtained with the product in question, alone or blended with lubricants if highly abrasive. ^b^ Determines particle size, in accordance with the percentages of the different particle size fractions obtained by applying Equation (1).

**Table 2:** Parameters, radius values, and incidence factors for Linezolid Glenmarck batch: GL-1, GL-2, and GL-3.

		GL-1				GL-2				GL-3			
Parameter	Symbol	R1	R2	R3	$\bar{R}$	R1	R2	R3	$\bar{R}$	R1	R2	R3	$\bar{R}$
Bulk density	Da	4.29	4.09	4.16	4.18	4.13	4.18	4.21	4.17	4.24	4.13	4.16	4.18
Tapped density	Dc	5.48	5.34	5.38	5.40	5.48	5.42	5.59	5.50	5.58	5.42	5.44	5.48
Interparticle porosity	Ie	4.22	4.77	4.54	4.51	4.97	4.56	4.89	4.81	4.72	4.80	4.71	4.75
Carr index	IC	4.34	4.68	4.54	4.52	4.93	4.58	4.94	4.81	4.80	4.76	4.71	4.76
Cohesion index	Icd	1.59	1.83	2.13	1.85	1.92	1.76	1.85	1.84	1.54	1.76	1.62	1.64
Hausner ratio	IH	8.61	8.47	8.53	8.54	8.37	8.52	8.36	8.41	8.42	8.44	8.46	8.44
Angle of repose	(α)	1.97	1.27	1.79	1.68	1.17	1.33	1.30	1.27	1.33	1.55	1.24	1.37
Flowability	t″	0.00	0.00	0.00	0.00	0.00	0.00	0.00	0.00	0.00	0.00	0.00	0.00
Loss on drying	%HR	9.83	9.78	9.76	9.79	9.53	9.78	9.67	9.66	9.65	9.70	9.65	9.67
Hygroscopicity	%H	10.00	9.99	9.99	9.99	10.00	10.00	9.99	9.99	10.00	10.00	9.99	9.99
Particles < 50 µm	%Pf	7.52	7.45	7.74	7.57	7.72	7.75	7.57	7.68	7.62	7.90	7.80	7.78
Homogeneity index	(Iθ)	4.50	4.40	3.65	4.18	4.10	3.60	4.40	4.03	4.60	3.45	3.90	3.98
Parametric index		0.42	0.42	0.42	0.42	0.42	0.42	0.42	0.42	0.42	0.42	0.42	0.42
Parametric profile (radius mean)		5.20	5.17	5.18	5.18	5.19	5.12	5.23	5.18	5.21	5.16	5.14	5.17
Good compression index (IGC)		4.95	4.92	4.94	4.94	4.94	4.88	4.98	4.93	4.96	4.91	4.89	4.92

**Table 3:** Parameters, radius values, and incidence factors for the Linezolid USV batch: US-1, US-2, and US-3.

		**US-1**				**US-2**				**US-2**			
**Parameter**	**Symbol**	**R1**	**R2**	**R3**	$\bar{R}$	**R1**	**R2**	**R3**	$\bar{R}$	**R1**	**R2**	**R3**	$\bar{R}$
Bulk density	Da	2.59	2.49	2.63	2.57	2.51	2.48	2.53	2.51	2.47	2.54	2.58	2.53
Tapped density	Dc	3.56	3.41	3.56	3.51	3.44	3.34	3.48	3.42	3.38	3.63	3.56	3.52
Interparticle porosity	Ie	8.77	9.03	8.28	8.69	8.98	8.65	8.99	8.87	9.08	9.85	8.89	9.28
Carr index	IC	5.45	5.40	5.22	5.36	5.41	5.15	5.46	5.34	5.38	6.01	5.51	5.63
Cohesion index	Icd	0.00	0.00	0.00	0.00	0.00	0.00	0.00	0.00	0.00	0.00	0.00	0.00
Hausner ratio	IH	8.13	8.15	8.23	8.17	8.15	8.27	8.12	8.18	8.16	7.85	8.10	8.04
Angle of repose	(α)	0.51	0.73	0.84	0.69	1.04	0.74	1.34	1.04	0.90	1.18	0.74	0.94
Flowability	t″	0.00	0.00	0.00	0.00	0.00	0.00	0.00	0.00	0.00	0.00	0.00	0.00
Loss on drying	%HR	9.67	9.65	9.60	9.64	9.62	9.61	9.57	9.60	9.69	9.52	9.61	9.61
Hygroscopicity	%H	9.96	9.98	10.00	9.98	10.00	10.00	10.00	10.00	10.00	10.00	9.99	10.00
Particles < 50 µm	%Pf	9.18	9.29	9.34	9.27	9.48	9.59	9.30	9.46	9.44	9.16	9.38	9.32
Homogeneity index	(Iθ)	9.65	9.40	8.95	9.33	9.25	8.45	8.20	8.63	9.00	8.75	8.00	8.58
Parametric index		0.58	0.58	0.58	0.58	0.58	0.58	0.58	0.58	0.58	0.58	0.58	0.58
Parametric profile (radius mean)		5.62	5.63	5.55	5.60	5.66	5.52	5.58	5.59	5.63	5.71	5.53	5.62
Good compression index (IGC)		5.35	5.36	5.29	5.33	5.38	5.26	5.32	5.32	5.36	5.43	5.26	5.35

**Table 4:** Parameters, radius values, and incidence factors for the Linezolid UQUIFA batch: UQ-1, UQ-2, and UQ-3.

		UQ-1				UQ-2				UQ-3			
Parameter	Symbol	R1	R2	R3	$\bar{R}$	R1	R2	R3	$\bar{R}$	R1	R2	R3	$\bar{R}$
Bulk density	Da	2.36	2.32	2.40	2.36	2.26	2.29	2.33	2.29	2.28	2.30	2.34	2.31
Tapped density	Dc	2.72	2.61	2.68	2.67	2.57	2.63	2.69	2.63	2.62	2.54	2.68	2.61
Interparticle porosity	Ie	4.67	3.99	3.63	4.10	4.45	4.70	4.79	4.65	4.74	3.42	4.52	4.23
Carr index	IC	2.65	2.22	2.09	2.32	2.41	2.59	2.68	2.56	2.60	1.89	2.54	2.34
Cohesion index	Icd	0.00	0.00	0.00	0.00	0.00	0.00	0.00	0.00	0.00	0.00	0.00	0.00
Hausner ratio	IH	9.24	9.38	9.42	9.34	9.31	9.26	9.23	9.27	9.25	9.48	9.27	9.34
Angle of repose	(α)	1.27	0.64	1.55	1.16	0.95	0.65	0.84	0.81	0.49	0.97	0.47	0.64
Flowability	t″	0.00	0.00	0.00	0.00	0.00	0.00	0.00	0.00	0.00	0.00	0.00	0.00
Loss on drying	%HR	9.45	9.83	9.11	9.46	8.86	9.20	9.91	9.32	9.12	9.78	9.65	9.52
Hygroscopicity	%H	9.97	9.97	9.98	9.98	9.97	9.98	9.99	9.98	10.00	9.97	9.98	9.98
Particles < 50 µm	%Pf	2.96	3.55	3.76	3.42	2.49	3.15	3.05	2.90	3.13	3.54	2.78	3.15
Homogeneity index	(Iθ)	2.20	2.15	2.40	2.25	2.70	2.25	2.40	2.45	2.85	2.50	2.40	2.58
Parametric index		0.25	0.25	0.25	0.25	0.25	0.25	0.25	0.25	0.25	0.25	0.25	0.25
Parametric profile (radius mean)		3.96	3.89	3.92	3.92	3.83	3.89	3.99	3.90	3.92	3.87	3.89	3.89
Good compression index (IGC)		3.77	3.70	3.73	3.73	3.65	3.70	3.80	3.72	3.73	3.68	3.70	3.70

**Table 5:** Statistical parameters for each source of Linezolid (pilot-scale batches).

Parameter	Glenmarck				USV				UQUIFA			
	$\bar{x}$	S^2^	Sn − 1	CV%	$\bar{x}$	S2	Sn − 1	CV%	$\bar{x}$	S^2^	Sn − 1	CV%
Da	4.1767	0.0038	0.0616	1.4759	2.5356	0.0030	0.0548	2.1612	2.3200	0.0019	0.0433	1.8664
Dc	5.4589	0.0071	0.0840	1.5393	3.4844	0.0097	0.0985	2.8269	2.6378	0.0035	0.0595	2.2570
Ie	4.6871	0.0498	0.2232	4.7630	8.9466	0.1772	0.4209	4.7046	4.3239	0.2645	0.5143	11.8939
IC	4.6966	0.0365	0.1912	4.0702	5.4425	0.0577	0.2401	4.4119	2.4062	0.0771	0.2777	11.5428
Icd ^a^	1.7767	0.0338	0.1839	10.3527	0.0000	0.0000	0.0000	0.0000	0.0000	0.0000	0.0000	0.0000
IH	8.4646	0.0066	0.0812	0.9599	8.1290	0.0134	0.1159	1.4255	9.3149	0.0079	0.0887	0.9525
(α)	1.4387	0.0758	0.2753	19.1339	0.8913	0.0660	0.2568	28.8146	0.8706	0.1319	0.3632	41.7191
t″ ^b^	0.0000	0.0000	0.0000	NA ^c^	0.0000	0.0000	0.0000	NA ^c^	0.0000	0.0000	0.0000	NA ^c^
%HR	9.7052	0.0087	0.0933	0.9610	9.6142	0.0026	0.0507	0.5270	9.4340	0.1429	0.3780	4.0072
%H	9.9928	0.0000	0.0044	0.0441	9.9904	0.0002	0.0152	0.1524	9.9775	0.0001	0.0084	0.0843
%Pf	7.6750	0.0217	0.1472	1.9173	9.3496	0.0192	0.1384	1.4802	3.1572	0.1623	0.4029	12.7616
(Iθ)	4.0667	0.1869	0.4323	10.6301	8.8500	0.3063	0.5534	6.2531	2.4278	0.0526	0.2293	9.4440
PP	5.1782	0.0011	0.0338	0.6527	5.6028	0.0037	0.0611	1.0904	3.9058	0.0023	0.0483	1.2372
IGC	4.9297	0.0010	0.0322	0.6527	5.3339	0.0034	0.0582	1.0904	3.7184	0.0021	0.0460	1.2372

^a^ All values for USV and UQUIFA sources were found to be the same, so they could not be analyzed statistically. ^b^ All values for this parameter were found to be the same, so they could not be analyzed statistically. ^c^ Not applicable.

**Table 6:** Statistical tests carried out on samples studied of different sources (pilot-scale batches).

Parameter	Glenmarck			USV			UQUIFA		
	Variance Check	ANOVA		Variance Check	ANOVA		Variance Check	ANOVA	
	Levene’s	F-Ratio	*p*-Value	Levene’s	F-Ratio	*p*-Value	Levene’s	F-Ratio	*p*-Value
Da	0.5787	0.01	0.9934	0.6164	1.03	0.4114	0.9302	2.97	0.1267
Dc	0.9796	1.19	0.3676	0.8220	0.97	0.4317	0.9460	0.66	0.5524
Ie	0.4394	1.74	0.2529	0.6907	1.83	0.2401	0.6047	0.91	0.4512
IC	0.6607	3.02	0.1236	0.6452	1.60	0.2779	0.7681	0.61	0.5728
Icd ^a^	0.3422	1.39	0.3184	NA ^c^	NA ^c^	NA ^c^	NA ^c^	NA ^c^	NA ^c^
IH	0.6493	2.73	0.1438	0.0192	1.60	0.2783	0.7767	0.61	0.5728
(α)	0.4561	2.47	0.1649	0.7526	1.71	0.2589	0.6188	1.87	0.2331
t″ ^b^	NA ^c^	NA ^c^	NA ^c^	NA ^c^	NA ^c^	NA ^c^	NA ^c^	NA ^c^	NA ^c^
%HR	0.2506	2.70	0.1454	0.3694	0.45	0.6589	0.8418	0.17	0.8487
%H	1.0000	0.33	0.7290	0.1537	2.38	0.1729	0.5748	0.20	0.8249
%Pf	0.6631	4.74	0.0582	0.8115	1.65	0.2680	0.9751	1.41	0.3149
(Iθ)	0.9055	0.14	0.8752	0.9077	2.27	0.1846	0.8220	2.01	0.2143
PP	0.4996	0.04	0.9590	0.6293	0.18	0.8389	0.4390	023	0.7989
IGC	0.4996	0.04	0.9590	0.6293	0.18	0.8389	0.4390	0.23	0.7989

^a^ All values for USV and UQUIFA sources were found to be the same, so they could not be analyzed statistically. ^b^ All values for this parameter were found to be the same, so they could not be analyzed statistically. ^c^ Not applicable.

**Table 7:** Values of D_10_, D_50_, D_90_, and F’ of all the sources.

Source	D_10_ (µm)	D_50_ (µm)	D_90_ (µm)	F’
Glenmarck	13.053	59.635	163.493	12.525
USV	4.715	13.396	51.339	10.888
UQUIFA	2.813	8.865	30.443	10.822

**Table 8:** Parameters, radius values, and incidence factors for Linezolid Glenmarck batch: GL-4, GL-5, and GL-6.

		GL-4				GL-5				GL-6			
Parameter	Symbol	R1	R2	R3	$\bar{R}$	R1	R2	R3	$\bar{R}$	R1	R2	R3	$\bar{R}$
Bulk density	Da	4.14	4.19	4.23	4.19	4.24	4.16	4.18	4.19	4.20	4.23	4.25	4.23
Tapped density	Dc	5.42	5.44	5.41	5.42	5.58	5.44	5.46	5.49	5.56	5.44	5.57	5.52
Interparticle porosity	Ie	4.75	4.57	4.30	4.54	4.72	4.71	4.67	4.70	4.85	4.38	4.65	4.63
Carr index	IC	4.72	4.60	4.36	4.56	4.80	4.71	4.69	4.73	4.89	4.44	4.74	4.69
Cohesion index	Icd	2.08	1.75	2.13	1.99	2.01	2.03	1.80	1.94	2.11	1.96	1.76	1.94
Hausner ratio	IH	8.45	8.51	8.61	8.52	8.42	8.46	8.47	8.45	8.38	8.57	8.45	8.47
Angle of repose	(α)	1.53	1.22	1.98	1.58	1.45	1.56	1.01	1.34	1.33	2.19	1.84	1.78
Flowability	t″	0.00	0.00	0.00	0.00	0.00	0.00	0.00	0.00	0.00	0.00	0.00	0.00
Loss on drying	%HR	9.58	9.79	9.78	9.72	9.66	9.77	9.67	9.70	9.65	9.70	9.75	9.70
Hygroscopicity	%H	9.99	9.99	10.00	9.99	9.99	10.00	10.00	9.99	9.99	10.00	9.99	9.99
Particles < 50 µm	%Pf	7.40	7.75	7.54	7.56	7.53	7.71	7.55	7.60	7.48	7.86	7.86	7.74
Homogeneity index	(Iθ)	4.50	4.40	4.15	4.35	4.15	3.95	4.50	4.20	4.10	4.15	3.65	3.97
Parametric index		0.42	0.42	0.42	0.42	0.42	0.42	0.42	0.42	0.42	0.42	0.42	0.42
Parametric profile (radius mean)		5.21	5.18	5.21	5.20	5.21	5.21	5.17	5.20	5.21	5.24	5.21	5.22
Good compression index (IGC)		4.96	4.94	4.96	4.95	4.96	4.96	4.92	4.95	4.96	4.99	4.96	4.97

**Table 9:** Parameters, radius values, and incidence factors for Linezolid USV batch: US-4, US-5, and US-6.

		US-4				US-5				US-6			
Parameter	Symbol	R1	R2	R3	$\bar{R}$	R1	R2	R3	$\bar{R}$	R1	R2	R3	$\bar{R}$
Bulk density	Da	2.58	2.62	2.55	2.58	2.62	2.49	2.53	2.55	2.55	2.47	2.58	2.53
Tapped density	Dc	3.56	3.57	3.65	3.59	3.56	3.41	3.57	3.51	3.59	3.47	3.62	3.56
Interparticle porosity	Ie	8.89	8.46	9.85	9.07	10.00	9.03	9.60	9.54	10.00	9.72	9.28	9.67
Carr index	IC	5.51	5.32	6.03	5.62	5.28	5.40	5.83	5.50	5.79	5.76	5.75	5.77
Cohesion index	Icd	0.00	0.00	0.00	0.00	0.00	0.00	0.00	0.00	0.00	0.00	0.00	0.00
Hausner ratio	IH	8.10	8.19	7.84	8.04	8.21	8.15	7.94	8.10	7.96	7.98	7.98	7.97
Angle of repose	(α)	0.72	1.19	0.96	0.96	0.70	1.38	0.94	1.01	0.76	1.10	0.96	0.94
Flowability	t″	0.00	0.00	0.00	0.00	0.00	0.00	0.00	0.00	0.00	0.00	0.00	0.00
Loss on drying	%HR	9.59	9.68	9.55	9.61	9.69	9.62	9.61	9.64	9.69	9.64	9.59	9.64
Hygroscopicity	%H	9.99	9.99	9.99	9.99	9.96	10.00	9.99	9.98	9.96	9.99	9.99	9.98
Particles < 50 µm	%Pf	9.40	9.28	9.39	9.36	9.59	9.38	9.46	9.47	9.48	9.44	9.46	9.46
Homogeneity index	(Iθ)	9.95	8.40	9.40	9.25	9.60	9.10	8.90	9.20	9.10	9.80	8.90	9.27
Parametric index		0.58	0.58	0.58	0.58	0.58	0.58	0.58	0.58	0.58	0.58	0.58	0.58
Parametric profile (radius mean)		5.69	5.56	5.77	5.67	5.77	5.66	5.70	5.71	5.74	5.78	5.67	5.73
Good compression index (IGC)		5.42	5.29	5.49	5.40	5.49	5.39	5.42	5.43	5.47	5.50	5.40	5.46

**Table 10:** Parameters, radius values, and incidence factors for Linezolid UQUIFA batch: UQ-4, UQ-5, and UQ-6.

		UQ-4				UQ-5				UQ-6			
Parameter	Symbol	R1	R2	R3	$\bar{R}$	R1	R2	R3	$\bar{R}$	R1	R2	R3	$\bar{R}$
Bulk density	Da	2.28	2.27	2.32	2.29	2.27	2.29	2.35	2.30	2.26	2.37	2.35	2.33
Tapped density	Dc	2.58	2.60	2.68	2.62	2.58	2.69	2.63	2.63	2.57	2.68	2.67	2.64
Interparticle porosity	Ie	4.25	4.66	4.83	4.58	4.41	5.41	3.78	4.53	4.45	4.07	4.25	4.25
Carr index	IC	2.33	2.54	2.69	2.52	2.40	2.97	2.13	2.50	2.41	2.31	2.40	2.37
Cohesion index	Icd	0.00	0.00	0.00	0.00	0.00	0.00	0.00	0.00	0.00	0.00	0.00	0.00
Hausner ratio	IH	9.34	9.27	9.22	9.28	9.32	9.13	9.40	9.28	9.31	9.35	9.32	9.33
Angle of repose	(α)	0.95	0.77	1.35	1.02	1.51	0.65	1.09	1.08	1.05	0.98	1.35	1.12
Flowability	t″	0.00	0.00	0.00	0.00	0.00	0.00	0.00	0.00	0.00	0.00	0.00	0.00
Loss on drying	%HR	9.69	9.83	9.47	9.66	9.26	9.33	9.92	9.50	9.48	9.77	9.65	9.63
Hygroscopicity	%H	9.97	9.98	9.98	9.97	9.97	9.98	9.99	9.98	9.99	9.99	9.98	9.98
Particles < 50 µm	%Pf	3.17	3.77	3.53	3.49	3.31	3.35	3.51	3.39	3.06	3.70	3.57	3.44
Homogeneity index	(Iθ)	2.62	2.15	2.40	2.39	2.17	2.85	2.50	2.51	1.85	2.40	2.60	2.28
Parametric index		0.25	0.25	0.25	0.25	0.25	0.25	0.25	0.25	0.25	0.25	0.25	0.25
Parametric profile (radius mean)		3.93	3.99	4.04	3.99	3.93	4.05	3.94	3.98	3.87	3.97	4.01	3.95
Good compression index (IGC)		3.74	3.80	3.84	3.79	3.74	3.86	3.75	3.79	3.68	3.78	3.82	3.76

**Table 11:** Statistical parameters for each source of Linezolid (industrial-scale batches).

Parameter	Glenmark				USV				UQUIFA			
	$\bar{x}$	S^2^	Sn − 1	CV%	$\bar{x}$	S2	Sn − 1	CV%	$\bar{x}$	S^2^	Sn − 1	CV%
Da	4.2022	0.0014	0.0380	0.9044	2.5544	0.0027	0.0522	2.0446	2.2967	0.0016	0.0406	1.7687
Dc	5.4798	0.0048	0.0692	1.2637	3.5556	0.0054	0.0735	2.0673	2.6244	0.0029	0.0536	2.0440
Ie	4.6226	0.0326	0.1805	3.9054	9.4257	0.2931	0.5414	5.7434	4.5299	0.1599	0.3998	8.8265
IC	4.6615	0.0284	0.1686	3.6175	5.6291	0.0677	0.2602	4.6221	2.4959	0.0452	0.2125	8.5151
Icd ^a^	1.9572	0.0226	0.1502	7.6747	0.0000	0.0000	0.0000	0.0000	0.0000	0.0000	0.0000	0.0000
IH	8.4798	0.0051	0.0713	0.8403	8.0395	0.0159	0.1260	1.5667	9.2863	0.0050	0.0705	0.7595
(α)	1.5681	0.1422	0.3771	24.0471	0.9672	0.0510	0.2259	23.3535	1.0722	0.0809	0.2844	26.5214
t″ ^b^	0.0000	0.0000	0.0000	NA ^c^	0.0000	0.0000	0.0000	NA ^c^	0.0000	0.0000	0.0000	NA ^c^
%HR	9.7051	0.0054	0.0737	0.7591	9.6291	0.0026	0.0508	0.5271	9.5498	0.0388	0.1970	2.0625
%H	9.9929	0.0000	0.0042	0.0416	9.9823	0.0003	0.0161	0.1617	9.9793	0.0001	0.0084	0.0841
%Pf	7.6316	0.0286	0.1692	2.2166	9.4299	0.0073	0.0852	0.9033	3.3907	0.0712	0.2667	7.8669
(Iθ)	4.1722	0.0744	0.2728	6.5396	9.2389	0.2436	0.4936	5.3423	2.3206	0.1190	0.3450	14.8681
PP	5.2061	0.0004	0.0211	0.4044	5.7043	0.0049	0.0700	1.2279	3.9621	0.0045	0.0672	1.6971
IGC	4.9562	0.0004	0.0200	0.4044	5.4305	0.0044	0.0667	1.2279	3.7720	0.0041	0.0640	1.6971

^a^ All values for USV and UQUIFA sources were found to be the same, so they could not be analyzed statistically. ^b^ All values for this parameter were found to be the same, so they could not be analyzed statistically. ^c^ Not applicable.

**Table 12:** Statistical tests carried out on the radius and incidence factor for Linezolid (industrial-scale batches).

Parameter	Glenmarck			USV			UQUIFA		
	Variance Check	ANOVA		Variance Check	ANOVA		Variance Check	ANOVA	
	Levene’s	F-Ratio	*p*-Value	Levene’s	F-Ratio	*p*-Value	Levene’s	F-Ratio	*p*-Value
Da	0.7996	0.94	0.4416	0.6164	1.03	0.4114	0.5661	1.53	0.2896
Dc	0.6709	2.12	0.2016	0.8220	0.97	0.4317	0.9883	0.16	0.8569
Ie	0.3084	0.54	0.6105	0.6907	1.83	0.2401	0.7606	1.30	0.3399
IC	0.4948	0.82	0.4851	0.6452	1.60	0.2779	0.6437	0.79	0.4942
Icd	0.8953	0.06	0.9439	0.0000	0.0000	0.0000	0.0000	0.0000	0.0000
IH	0.4831	0.81	0.4891	0.0192	1.60	0.2783	0.6413	0.82	0.4863
(α)	0.8686	1.08	0.3988	0.7526	1.71	0.2589	0.6436	0.08	0.9266
t″	0.0000	0.0000	0.0000	0.0000	0.0000	0.0000	0.0000	0.0000	0.0000
%HR	0.8063	0.04	0.9599	0.3694	0.45	0.6589	0.8310	3.85	0.0840
%H	1.0000	0.33	0.7290	0.1537	2.38	0.1729	0.7290	1.40	0.3170
%Pf	0.8450	0.82	0.4838	0.8115	1.65	0.2680	0.7505	0.69	0.5372
(Iθ)	0.9056	1.81	0.82430	0.9077	2.27	0.1846	0.7299	0.07	0.9342
PP	0.9403	1.33	0.3345	0.6293	0.18	0.8389	0.8105	0.22	0.8089
IGC	0.9403	1.33	0.3345	0.6293	0.18	0.8389	0.8105	0.22	0.8089

**Table 13:** Statistical test carried out on the radius and incidence factor for Linezolid (pilot and industrial scale).

Parameter	Glenmarck				USV				UQUIFA			
	$\bar{x}$	S^2^	Sn − 1	CV%	$\bar{x}$	S^2^	Sn − 1	CV%	$\bar{x}$	S^2^	Sn − 1	CV%
Da	4.1894	0.0026	0.0514	1.2266	2.5450	0.0028	0.0528	2.0759	2.3083	0.0018	0.0425	1.8395
Dc	5.4693	0.0057	0.0755	1.3798	3.5200	0.0084	0.0919	2.6110	2.6311	0.0031	0.0554	2.1056
Ie	4.6549	0.0399	0.1997	4.2907	9.1861	0.2820	0.5311	5.7812	4.4269	0.2109	0.4593	10.3744
IC	4.6790	0.0309	0.1758	3.7571	5.5358	0.0682	0.2612	4.7177	2.4511	0.0597	0.2443	9.9675
Icd ^a^	1.8669	0.0352	0.1875	10.0448	0.0000	0.0000	0.0000	0.0000	0.0000	0.0000	0.0000	0.0000
IH	8.4722	0.0056	0.0745	0.8798	8.0842	0.0159	0.1261	1.5600	9.3006	0.0063	0.0791	0.8509
(α)	1.5034	0.1070	0.3271	21.7591	0.9292	0.0566	0.2378	25.5959	0.9714	0.1109	0.3330	34.2803
t″ ^b^	0.0000	0.0000	0.0000	0.0000	0.0000	0.0000	0.0000	0.0000	0.0000	0.0000	0.0000	0.0000
%HR	9.7052	0.0066	0.0815	0.8401	9.6217	0.0025	0.0498	0.5174	9.4919	0.0891	0.2984	3.1440
%H	9.9928	0.0000	0.0042	0.0416	9.9864	0.0002	0.0158	0.1580	9.9784	0.0001	0.0082	0.0822
%Pf	7.6533	0.0242	0.1554	2.0307	9.3898	0.0141	0.1189	1.2662	3.2740	0.1243	0.3526	10.7691
(Iθ)	4.1194	0.1259	0.3549	8.6142	9.0444	0.2988	0.5466	6.0437	2.3742	0.0838	0.2895	12.1931
PP	5.1922	0.0010	0.0308	0.5941	5.6536	0.0068	0.0824	1.4578	3.9340	0.0041	0.0638	1.6209
IGC	4.9429	0.0009	0.0294	0.5941	5.3822	0.0062	0.0785	1.4578	3.7452	0.0037	0.0607	1.6209

^a^ All values for USV and UQUIFA sources were found to be the same, so they could not be analyzed statistically. ^b^ All values for this parameter were found to be the same, so they could not be analyzed statistically.

**Table 14:** Statistical parameters for each source of Linezolid (pilot and industrial batches).

Parameter	Glenmark			USV			UQUIFA		
	Variance Check	ANOVA		Variance Check	ANOVA		Variance Check	ANOVA	
	Levene’s	F-Ratio	*p*-Value	Levene’s	F-Ratio	*p*-Value	Levene’s	F-Ratio	*p*-Value
Da	0.6708	0.35	0.8704	0.6164	1.03	0.4114	0.8653	2.12	0.1326
Dc	0.9296	1.29	0.3302	0.8220	0.97	0.4317	0.9994	0.37	0.8566
Ie	0.5013	1.05	0.4352	0.6907	1.83	0.2401	0.8458	1.03	0.4440
IC	0.7783	1.49	0.2633	0.6452	1.60	0.2779	0.8814	0.64	0.6726
Icd	0.8148	1.56	0.2436	0.0000	0.0000	0.0000	0.0000	0.0000	0.0000
IH	0.7643	1.38	0.2996	0.0192	1.60	0.2783	0.8804	0.67	0.6561
(α)	0.7204	1.33	0.3160	0.7526	1.71	0.2589	0.8343	1.15	0.3867
t″	0.0000	0.0000	0.0000	0.0000	0.0000	0.0000	0.0000	0.0000	0.0000
%HR	0.7381	1.03	0.4425	0.3694	0.45	0.6589	0.6071	0.58	0.7154
%H	1.0000	0.30	0.9036	0.1537	2.38	0.1729	0.7708	0.75	0.6017
%Pf	0.9767	1.01	0.4513	0.8115	1.65	0.2680	0.9621	1.37	0.3018
(Iθ)	0.8640	0.45	0.8044	0.9077	2.27	0.1846	0.7597	0.49	0.7785
PP	0.6285	1.06	0.4264	0.6293	0.18	0.8389	0.7708	0.85	0.5406
IGC	0.6285	1.06	0.4264	0.6293	0.18	0.8389	0.7708	0.85	0.5406

**Table 15:** Comparison of the parameters of different sources (pilot- and industrial-scale batches).

	PILOT SCALE			INDUSTRIAL SCALE			AVERAGE		
	Glenmarck	USV	UQUIFA	Glenmarck	USV	UQUIFA	Glenmarck	USV	UQUIFA
Da	4.18	2.54	2.32	4.20	2.55	2.30	4.19	2.55	2.31
Dc	5.46	3.48	2.64	5.48	3.56	2.62	5.47	3.52	2.63
Dimensions	4.82	3.01	2.48	4.84	3.06	2.46	4.83	3.03	2.47
Ie	4.69	8.95	4.32	4.62	9.43	4.53	4.65	9.19	4.43
IC	4.70	5.44	2.41	4.66	5.63	2.50	4.68	5.54	2.45
Icd	1.78	0.00	0.00	1.96	0.00	0.00	1.87	0.00	0.00
Compressibility	3.72	4.80	2.24	3.75	5.02	2.34	3.73	4.91	2.29
IH	8.46	8.13	9.31	8.48	8.04	9.29	8.47	8.08	9.30
(α)	1.44	0.89	0.87	1.57	0.97	1.07	1.50	0.93	0.97
t″	0.00	0.00	0.00	0.00	0.00	0.00	0.00	0.00	0.00
Flowability	3.30	3.01	3.40	3.35	3.00	3.45	3.33	3.00	3.42
%HR	9.71	9.61	9.43	9.71	9.63	9.55	9.71	9.62	9.49
%H	9.99	9.99	9.98	9.99	9.98	9.98	9.99	9.99	9.98
Lubricity/Stability	9.85	9.80	9.71	9.85	9.81	9.76	9.85	9.80	9.74
%Pf	7.68	9.35	3.16	7.63	9.43	3.39	7.65	9.39	3.27
(Iθ)	4.07	8.85	2.43	4.17	9.24	2.32	4.12	9.04	2.37
Lubricity/Dosage	5.87	9.10	2.79	5.90	9.33	2.86	5.89	9.22	2.82
IP	0.42	0.58	0.25	0.42	0.58	0.25	0.42	0.58	0.25
PP	5.18	5.60	3.91	5.21	5.70	3.96	5.19	5.65	3.93
IGC	4.93	5.33	3.72	4.96	5.43	3.77	4.94	5.38	3.75

**Table 16:** Statistical tests carried out on the radius and incidence factor for Linezolid (pilot and industrial scale).

Parameter	Glenmarck vs. USV			Glenmarck vs. UQUIFA			USV vs. UQUIFA		
	Variance Check	ANOVA		Variance Check	ANOVA		Variance Check	ANOVA	
	Levene’s	F-Ratio	*p*-Value	Levene’s	F-Ratio	*p*-Value	Levene’s	F-Ratio	*p*-Value
Da	0.4721	5856.96	0.0000	0.9278	10560.95	0.0000	0.5012	136.61	0.0000
Dc	0.9043	3274.92	0.0000	0.8488	9589.69	0.0000	0.9595	942.29	0.0000
Ie	0.06167	637.01	0.0000	0.2215	0.39	0.5409	0.2738	475.03	0.0000
IC	0.2262	87.70	0.0000	0.8715	574.72	0.0000	0.3649	783.87	0.0000
Icd	NA	NA	NA	NA	NA	NA	NA	NA	NA
IH	0.1540	81.22	0.0000	0.8014	575.24	0.0000	0.1205	657.81	0.0000
(α)	0.2124	16.73	0.0009	0.4614	9.92	0.0062	0.5597	0.76	0.3959
t″	NA	NA	NA	NA	NA	NA	NA	NA	NA
%HR	0.2272	6.95	0.0180	0.3385	2.62	0.1812	0.0583	1.34	0.2640
%H	0.4867	3.90	0.0657	0.6607	18.00	0.0006	0.6414	0.38	0.5468
%Pf	0.1051	821.47	0.0000	0.1968	1631.90	0.0000	0.530178	4208.22	0.0000
(Iθ)	0.1317	726.41	0.0000	0.3837	159.09	0.0000	0.3912	1186.43	0.0000
PP	0.0524	417.68	0.0000	0.0613	2806.45	0.0000	0.9241	2898.47	0.0000
IGC	0.0524	417.68	0.0000	0.0613	2806.45	0.0000	0.9241	2898.47	0.0000

## Data Availability

Not applicable.

## Supplementary Materials

The following supporting information can be downloaded at: https://www.mdpi.com/article/10.3390/pharmaceutics15082034/s1, Table S1: Parameters, radius values, and incidence factors for Linezolid Glenmarck batch GL-1; Table S2: Parameters, radius values, and incidence factors for Linezolid Glenmarck batch GL-2; Table S3: Parameters, radius values, and incidence factors for Linezolid Glenmarck batch GL-3; Table S4: Parameters, radius values, and incidence factors for Linezolid USV batch US-1; Table S5: Parameters, radius values, and incidence factors for Linezolid USV batch US-2; Table S6: Parameters, radius values, and incidence factors for Linezolid USV batch US-3; Table S7: Parameters, radius values, and incidence factors for Linezolid UQUIFA batch UQ-1; Table S8: Parameters, radius values, and incidence factors for Linezolid USV batch UQ-2; Table S9: Parameters, radius values, and incidence factors for Linezolid USV batch UQ-3; Table S10: Parameters, radius values, and incidence factors for Linezolid Glenmarck batch GL-4; Table S11: Parameters, radius values, and incidence factors for Linezolid Glenmarck batch GL-5; Table S12: Parameters, radius values, and incidence factors for Linezolid Glenmarck batch GL-6; Table S13: Parameters, radius values, and incidence factors for Linezolid USV batch US-4; Table S14: Parameters, radius values, and incidence factors for Linezolid USV batch US-5; Table S15: Parameters, radius values, and incidence factors for Linezolid USV batch US-6; Table S16: Parameters, radius values, and incidence factors for Linezolid UQUIFA batch UQ-4; Table S17: Parameters, radius values, and incidence factors for Linezolid USV batch UQ-5; Table S18: Parameters, radius values, and incidence factors for Linezolid USV batch UQ-6; Table S19: Composition of the Linezolid tablets produced with direct compression technology, API batch: US-3; Table S20. Critical quality attributes of Linezolid tablets (Batch US 3.3), USV source obtained by direct compression.
