# Supplementary material for: SeDeM as a Tool to Validate Drug Substance Manufacturing Processes and Assess Scalability and Suitability for Direct Compression: Supplier Screening
Source: Pharmaceutics. 2023 Jul 28;15(8):2034. doi: 10.3390/pharmaceutics15082034 (PMC10457849; doi:10.3390/pharmaceutics15082034)
Supplement: Supplementary file 1 [file pharmaceutics-15-02034-s001.zip › pharmaceutics-2500850-supplementary.pdf]

Supplementary Information

SeDeM as a Tool to Validate Drug Substance Manufacturing Processes and Assess Scalability and Suitability for Direct Compression: Supplier Screening

Alba Figuera-Figuera [1], Marc Suñé-Pou [1,2,*], Pilar Pérez-Lozano [1,2], Encarna García-Montoya [1,2], Joaquim Amela-Navarro [1] and Josep M. Suñé-Negre [1,2]

[1] Pharmaceutical Technology and Physico-Chemical Department, Universitat de Barcelona, Av. Joan XXIII, 27-31, 08028 Barcelona, Spain; albafiguera099@gmail.com (A.F.-F.); perezlo@ub.edu (P.P.-L.); encarnagarcia@ub.edu (E.G.-M.); jamelan@ub.edu (J.A.-N.); jmsune@ub.edu (J.M.S.-N.)

[2] IDIBELL-UB Research Group, Pharmacotherapy, Pharmacogenomics and Pharmaceutical Technology, Avinguda Granvia, 199-203, 08908 L'Hospitalet de Llobregat, Spain

* Correspondence: marcsune@ub.edu

Table S1. Parameters, radius values, and incidence factors for Linezolid Glenmarck batch GL-1. Individual experimental values (v1,v2 and v3) and mean of experimental values (v), radius of different parameters after applying factor described in Table 1 (R1, R2 and R3) and mean value of radius (R). Parameter index (IP), Parameter profile index (IPP) and Good compression index (GCI).

**BATCH GL-1**											
**Incidence**	**Parameter**	**Symbol**	**Unit**	**v1**	**v2**	**v3**	**v**	**R1**	**R2**	**R3**	**R**
**Dimensions**	Bulk density	Da	g/ml	0,429	0,409	0,416	0,418	4,29	4,09	4,16	4,18
	Tapped density	Dc	g/ml	0,548	0,534	0,538	0,540	5,48	5,34	5,38	5,40
**Compressibility**	Interparticle porosity	Ie	(-)	0,506	0,572	0,545	0,541	4,22	4,77	4,54	4,51
	Carr Index	IC	%	21,715	23,408	22,677	22,600	4,34	4,68	4,54	4,52
	Cohesion index	Icd	N •	31,800	36,500	42,600	36,967	1,59	1,83	2,13	1,85
**Flowability**	Hausner Ratio	IH	(-)	1,277	1,306	1,293	1,292	8,61	8,47	8,53	8,54
	Angle of repose	(α)	º	40,146	43,668	41,036	41,617	1,97	1,27	1,79	1,68
	Flowability	t''	s	∞	∞	∞	∞	0,00	0,00	0,00	0,00
**Lubrication/stability**	Loss on drying	%HR	%	0,171	0,218	0,242	0,210	9,83	9,78	9,76	9,79
	Higroscopicity	%H	%	0,000	0,020	0,030	0,017	10,00	9,99	9,99	9,99
**Dosage/ lubrication**	Particles < 50 μ	%Pf	μ	12,419	12,774	11,300	12,164	7,52	7,45	7,74	7,57
	Homogenity Index	(Iθ)	-	0,009	0,009	0,007	0,008	4,50	4,40	3,65	4,18
							Parametric index (IP)	0,42	0,42	0,42	0,42
							Parametric profile (IPP)	5,20	5,17	5,18	5,18
							Good compression index (GCI)	4,95	4,92	4,94	4,94

Table S2. Parameters, radius values, and incidence factors for Linezolid Glenmarck batch GL-2. Individual experimental values (v1,v2 and v3) and mean of experimental values (v), radius of different parameters after applying factor described in Table 1 (R1, R2 and R3) and mean value of radius (R). Parameter index (IP), Parameter profile index (IPP) and Good compression index (GCI).

**BATCH GL-2**											
**Incidence**	**Parameter**	**Symbol**	**Unit**	**v1**	**v2**	**v3**	**v**	**R1**	**R2**	**R3**	**R**
**Dimensions**	Bulk density	Da	g/ml	0,413	0,418	0,421	0,417	4,13	4,18	4,21	4,17
	Tapped density	Dc	g/ml	0,548	0,542	0,559	0,550	5,48	5,42	5,59	5,50
**Compressibility**	Interparticle porosity	Ie	(-)	0,596	0,547	0,586	0,577	4,97	4,56	4,89	4,81
	Carr Index	IC	%	24,635	22,878	24,687	24,067	4,93	4,58	4,94	4,81
	Cohesion index	Icd	N ·	38,400	35,200	37,000	36,867	1,92	1,76	1,85	1,84
**Flowability**	Hausner Ratio	IH	(-)	1,327	1,297	1,328	1,317	8,37	8,52	8,36	8,41
	Angle of repose	(α)	º	44,128	43,367	43,490	43,662	1,17	1,33	1,30	1,27
	Flowability	t''	s	∞	∞	∞	∞	0,00	0,00	0,00	0,00
**Lubrication/stability**	Loss on drying	%HR	%	0,475	0,219	0,328	0,341	9,53	9,78	9,67	9,66
	Higroscopicity	%H	%	0,010	0,010	0,020	0,013	10,00	10,00	9,99	9,99
**Dosage/ lubrication**	Particles < 50 μ	%Pf	μ	11,377	11,233	12,169	11,593	7,72	7,75	7,57	7,68
	Homogenity Index	(Iθ)	-	0,008	0,007	0,009	0,008	4,10	3,60	4,40	4,03
					Parametric index (IP)			0,42	0,42	0,42	0,42
					Parametric profile (IPP)			5,19	5,12	5,23	5,18
					Good compression index (GCI)			4,94	4,88	4,98	4,93

Table S3. Parameters, radius values, and incidence factors for Linezolid Glenmarck batch GL-3. Individual experimental values (v1,v2 and v3) and mean of experimental values (v), radius of different parameters after applying factor described in Table 1 (R1, R2 and R3) and mean value of radius (R). Parameter index (IP), Parameter profile index (IPP) and Good compression index (GCI).

**BATCH GL-3**											
**Incidence**	**Parameter**	**Symbol**	**Unit**	**v1**	**v2**	**v3**	**v**	**R1**	**R2**	**R3**	**R**
**Dimensions**	Bulk density	Da	g/ml	0,424	0,413	0,416	0,418	4,24	4,13	4,16	4,18
	Tapped density	Dc	g/ml	0,558	0,542	0,544	0,548	5,58	5,42	5,44	5,48
**Compressibility**	Interparticle porosity	Ie	(-)	0,566	0,576	0,566	0,570	4,72	4,80	4,71	4,75
	Carr Index	IC	%	24,014	23,801	23,529	23,781	4,80	4,76	4,71	4,76
	Cohesion index	Icd	N •	30,800	35,200	32,300	32,767	1,54	1,76	1,62	1,64
**Flowability**	Hausner Ratio	IH	(-)	1,316	1,312	1,308	1,312	8,42	8,44	8,46	8,44
	Angle of repose	(α)	º	43,369	42,235	43,821	43,142	1,33	1,55	1,24	1,37
	Flowability	t''	s	∞	∞	∞	∞	0,00	0,00	0,00	0,00
**Lubrication/stability**	Loss on drying	%HR	%	0,355	0,299	0,346	0,333	9,65	9,70	9,65	9,67
	Higroscopicity	%H	%	0,010	0,010	0,020	0,013	10,00	10,00	9,99	9,99
**Dosage/ lubrication**	Particles < 50 µ	%Pf	µ	11,876	10,485	10,992	11,118	7,62	7,90	7,80	7,78
	Homogenity Index	(Iθ)	-	0,009	0,007	0,008	0,008	4,60	3,45	3,90	3,98
				Parametric index (IP)				0,42	0,42	0,42	0,42
				Parametric profile (IPP)				5,21	5,16	5,14	5,17
				Good compression index (GCI)				4,96	4,91	4,89	4,92

Table S4. Parameters, radius values, and incidence factors for Linezolid USV batch US-1. Individual experimental values (v1,v2 and v3) and mean of experimental values (v), radius of different parameters after applying factor described in Table 1 (R1, R2 and R3) and mean value of radius (R). Parameter index (IP), Parameter profile index (IPP) and Good compression index (GCI).

BATCH US-1											
**Incidence**	**Parameter**	**Symbol**	**Unit**	**v1**	**v2**	**v3**	**v**	**R1**	**R2**	**R3**	**R**
**Dimensions**	Bulk density	Da	g/ml	0,259	0,249	0,263	0,257	2,59	2,49	2,63	2,57
	Tapped density	Dc	g/ml	0,356	0,341	0,356	0,351	3,56	3,41	3,56	3,51
**Compressibility**	Interparticle porosity	Ie	(-)	1,052	1,084	0,993	1,043	8,77	9,03	8,28	8,69
	Carr Index	IC	%	27,247	26,979	26,124	26,783	5,45	5,40	5,22	5,36
	Cohesion index	Icd	N •	0	0	0	0,000	0,00	0,00	0,00	0,00
**Flowability**	Hausner Ratio	IH	(-)	1,375	1,369	1,354	1,366	8,13	8,15	8,23	8,17
	Angle of repose	(α)	º	47,46	46,341	45,802	46,534	0,51	0,73	0,84	0,69
	Flowability	t''	s	∞	∞	∞	∞	0,00	0,00	0,00	0,00
**Lubrication/stability**	Loss on drying	%HR	%	0,333	0,352	0,402	0,362	9,67	9,65	9,60	9,64
	Higroscopicity	%H	%	0,09	0,04	0	0,043	9,96	9,98	10,00	9,98
**Dosage/ lubrication**	Particles < 50 μ	%Pf	μ	4,096	3,568	3,316	3,660	9,18	9,29	9,34	9,27
	Homogenity Index	(Iθ)	-	0,0193	0,0188	0,0179	0,019	9,65	9,40	8,95	9,33
					Parametric index (IP)			0,58	0,58	0,58	0,58
					Parametric profile (IPP)			5,62	5,63	5,55	5,60
					Good compression index (GCI)			5,35	5,36	5,29	5,33

Table S5. Parameters, radius values, and incidence factors for Linezolid USV batch US-2. Individual experimental values (v1,v2 and v3) and mean of experimental values (v), radius of different parameters after applying factor described in Table 1 (R1, R2 and R3) and mean value of radius (R). Parameter index (IP), Parameter profile index (IPP) and Good compression index (GCI).

BATCH US-2											
**Incidence**	**Parameter**	**Symbol**	**Unit**	**v1**	**v2**	**v3**	**v**	**R1**	**R2**	**R3**	**R**
**Dimensions**	Bulk density	Da	g/ml	0,251	0,248	0,253	0,251	2,51	2,48	2,53	2,51
	Tapped density	Dc	g/ml	0,344	0,334	0,348	0,342	3,44	3,34	3,48	3,42
**Compressibility**	Interparticle porosity	Ie	(-)	1,077	1,038	1,079	1,065	8,98	8,65	8,99	8,87
	Carr Index	IC	%	27,035	25,749	27,299	26,694	5,41	5,15	5,46	5,34
	Cohesion index	Icd	N •	0	0	0	0,000	0,00	0,00	0,00	0,00
**Flowability**	Hausner Ratio	IH	(-)	1,371	1,347	1,375	1,364	8,15	8,27	8,12	8,18
	Angle of repose	(α)	º	44,805	46,288	43,285	44,793	1,04	0,74	1,34	1,04
	Flowability	t''	s	∞	∞	∞	∞	0,00	0,00	0,00	0,00
**Lubrication/stability**	Loss on drying	%HR	%	0,38	0,392	0,432	0,401	9,62	9,61	9,57	9,60
	Higroscopicity	%H	%	0	0,01	0,002	0,004	10,00	10,00	10,00	10,00
**Dosage/ lubrication**	Particles < 50 μ	%Pf	μ	2,612	2,054	3,491	2,719	9,48	9,59	9,30	9,46
	Homogenity Index	(Iθ)	-	0,019	0,017	0,016	0,017	9,25	8,45	8,20	8,63
							Parametric index (IP)	0,58	0,58	0,58	0,58
							Parametric profile (IPP)	5,66	5,52	5,58	5,59
							Good compression index (GCI)	5,38	5,26	5,32	5,32

Table S6. Parameters, radius values, and incidence factors for Linezolid USV batch US-3. Individual experimental values (v1,v2 and v3) and mean of experimental values (v), radius of different parameters after applying factor described in Table 1 (R1, R2 and R3) and mean value of radius (R). Parameter index (IP), Parameter profile index (IPP) and Good compression index (GCI).

BATCH US-3											
**Incidence**	**Parameter**	**Symbol**	**Unit**	**v1**	**v2**	**v3**	**v**	**R1**	**R2**	**R3**	**R**
**Dimensions**	Bulk density	Da	g/ml	0,247	0,254	0,258	0,253	2,47	2,54	2,58	2,53
	Tapped density	Dc	g/ml	0,338	0,363	0,356	0,352	3,38	3,63	3,56	3,52
**Compressibility**	Interparticle porosity	Ie	(-)	1,090	1,182	1,067	1,113	9,08	9,85	8,89	9,28
	Carr Index	IC	%	26,923	30,028	27,528	28,160	5,38	6,01	5,51	5,63
	Cohesion index	Icd	N ·	0	0	0	0,000	0,00	0,00	0,00	0,00
**Flowability**	Hausner Ratio	IH	(-)	1,368	1,429	1,380	1,392	8,16	7,85	8,10	8,04
	Angle of repose	(α)	º	45,476	44,122	46,312	45,303	0,90	1,18	0,74	0,94
	Flowability	t''	s	∞	∞	∞	∞	0,00	0,00	0,00	0,00
**Lubrication/stability**	Loss on drying	%HR	%	0,311	0,478	0,392	0,394	9,69	9,52	9,61	9,61
	Higroscopicity	%H	%	0	0	0,03	0,010	10,00	10,00	9,99	10,00
**Dosage/ lubrication**	Particles < 50 μ	%Pf	μ	2,808	4,2	3,124	3,377	9,44	9,16	9,38	9,32
	Homogenity Index	(Iθ)	-	0,018	0,0175	0,016	0,017	9,00	8,75	8,00	8,58
					Parametric index (IP)			0,58	0,58	0,58	0,58
					Parametric profile (IPP)			5,63	5,71	5,53	5,62
					Good compression index (GCI)			5,36	5,43	5,26	5,35

Table S7. Parameters, radius values, and incidence factors for Linezolid UQUIFA batch UQ-1. Individual experimental values (v1,v2 and v3) and mean of experimental values (v), radius of different parameters after applying factor described in Table 1 (R1, R2 and R3) and mean value of radius (R). Parameter index (IP), Parameter profile index (IPP) and Good compression index (GCI).

BATCH UQ-1											
**Incidence**	**Parameter**	**Symbol**	**Unit**	**v1**	**v2**	**v3**	**v**	**R1**	**R2**	**R3**	**R**
**Dimensions**	Bulk density	Da	g/ml	0,236	0,232	0,24	0,236	2,36	2,32	2,40	2,36
	Tapped density	Dc	g/ml	0,272	0,261	0,268	0,267	2,72	2,61	2,68	2,67
**Compressibility**	Interparticle porosity	Ie	(-)	0,561	0,479	0,435	0,492	4,67	3,99	3,63	4,10
	Carr Index	IC	%	13,235	11,111	10,448	11,598	2,65	2,22	2,09	2,32
	Cohesion index	Icd	N ·	0	0	0	0,000	0,00	0,00	0,00	0,00
**Flowability**	Hausner Ratio	IH	(-)	1,153	1,125	1,117	1,131	9,24	9,38	9,42	9,34
	Angle of repose	(α)	º	43,628	46,81	42,236	44,225	1,27	0,64	1,55	1,16
	Flowability	t''	s	∞	∞	∞	∞	0,00	0,00	0,00	0,00
**Lubrication/stability**	Loss on drying	%HR	%	0,555	0,166	0,889	0,537	9,45	9,83	9,11	9,46
	Higroscopicity	%H	%	0,057	0,052	0,04	0,050	9,97	9,97	9,98	9,98
**Dosage/ lubrication**	Particles < 50 µ	%Pf	µ	35,188	32,268	31,185	32,880	2,96	3,55	3,76	3,42
	Homogenity Index	(Iθ)	-	0,0044	0,0043	0,0048	0,005	2,20	2,15	2,40	2,25
					Parametric index (IP)			0,25	0,25	0,25	0,25
					Parametric profile (IPP)			3,96	3,89	3,92	3,92
					Good compression index (GCI)			3,77	3,70	3,73	3,73

Table S8. Parameters, radius values, and incidence factors for Linezolid USV batch UQ-2. Individual experimental values (v1,v2 and v3) and mean of experimental values (v), radius of different parameters after applying factor described in Table 1 (R1, R2 and R3) and mean value of radius (R). Parameter index (IP), Parameter profile index (IPP) and Good compression index (GCI).

**BATCH UQ-2**											
**Incidence**	**Parameter**	**Symbol**	**Unit**	**v1**	**v2**	**v3**	**v**	**R1**	**R2**	**R3**	**R**
**Dimensions**	Bulk density	Da	g/ml	0,226	0,229	0,233	0,229	2,26	2,29	2,33	2,29
	Tapped density	Dc	g/ml	0,257	0,263	0,269	0,263	2,57	2,63	2,69	2,63
**Compressibility**	Interparticle porosity	Ie	(-)	0,534	0,565	0,574	0,558	4,45	4,70	4,79	4,65
	Carr Index	IC	%	12,062	12,928	13,383	12,791	2,41	2,59	2,68	2,56
	Cohesion index	Icd	N •	0	0	0	0,000	0,00	0,00	0,00	0,00
**Flowability**	Hausner Ratio	IH	(-)	1,137	1,148	1,155	1,147	9,31	9,26	9,23	9,27
	Angle of repose	(α)	º	45,265	46,774	45,782	45,940	0,95	0,65	0,84	0,81
	Flowability	t''	s	∞	∞	∞	∞	0,00	0,00	0,00	0,00
**Lubrication/stability**	Loss on drying	%HR	%	1,14	0,805	0,089	0,678	8,86	9,20	9,91	9,32
	Higroscopicity	%H	%	0,06	0,048	0,028	0,045	9,97	9,98	9,99	9,98
**Dosage/ lubrication**	Particles < 50 μ	%Pf	μ	37,534	34,262	34,734	35,510	2,49	3,15	3,05	2,90
	Homogenity Index	(Iθ)	-	0,0054	0,0045	0,0048	0,005	2,70	2,25	2,40	2,45
					Parametric index (IP)			0,25	0,25	0,25	0,25
					Parametric profile (IPP)			3,83	3,89	3,99	3,90
					Good compression index (GCI)			3,65	3,70	3,80	3,72

Table S9. Parameters, radius values, and incidence factors for Linezolid USV batch UQ-3. Individual experimental values (v1,v2 and v3) and mean of experimental values (v), radius of different parameters after applying factor described in Table 1 (R1, R2 and R3) and mean value of radius (R). Parameter index (IP), Parameter profile index (IPP) and Good compression index (GCI).

BATCH UQ-3											
**Incidence**	**Parameter**	**Symbol**	**Unit**	**v1**	**v2**	**v3**	**v**	**R1**	**R2**	**R3**	**R**
**Dimensions**	Bulk density	Da	g/ml	0,228	0,23	0,234	0,231	2,28	2,30	2,34	2,31
	Tapped density	Dc	g/ml	0,262	0,254	0,268	0,261	2,62	2,54	2,68	2,61
**Compressibility**	Interparticle porosity	Ie	(-)	0,569	0,411	0,542	0,507	4,74	3,42	4,52	4,23
	Carr Index	IC	%	12,977	9,449	12,687	11,704	2,60	1,89	2,54	2,34
	Cohesion index	Icd	N •	0	0	0	0,000	0,00	0,00	0,00	0,00
**Flowability**	Hausner Ratio	IH	(-)	1,149	1,104	1,145	1,133	9,25	9,48	9,27	9,34
	Angle of repose	(α)	º	47,558	45,129	47,639	46,775	0,49	0,97	0,47	0,64
	Flowability	t''	s	∞	∞	∞	∞	0,00	0,00	0,00	0,00
**Lubrication/stability**	Loss on drying	%HR	%	0,88	0,221	0,349	0,483	9,12	9,78	9,65	9,52
	Higroscopicity	%H	%	0,01	0,062	0,048	0,040	10,00	9,97	9,98	9,98
**Dosage/ lubrication**	Particles < 50 µ	%Pf	µ	34,339	32,304	36,111	34,251	3,13	3,54	2,78	3,15
	Homogenity Index	(Iθ)	-	0,0057	0,005	0,0048	0,005	2,85	2,50	2,40	2,58
					Parametric index (IP)			0,25	0,25	0,25	0,25
					Parametric profile (IPP)			3,92	3,87	3,89	3,89
					Good compression index (GCI)			3,73	3,68	3,70	3,70

Table S10. Parameters, radius values, and incidence factors for Linezolid Glenmarck batch GL-4. Individual experimental values (v1,v2 and v3) and mean of experimental values (v), radius of different parameters after applying factor described in Table 1 (R1, R2 and R3) and mean value of radius (R). Parameter index (IP), Parameter profile index (IPP) and Good compression index (GCI).

BATCH GL-4											
**Incidence**	**Parameter**	**Symbol**	**Unit**	**v1**	**v2**	**v3**	**v**	**R1**	**R2**	**R3**	**R**
**Dimensions**	Bulk density	Da	g/ml	0,414	0,419	0,423	0,419	4,14	4,19	4,23	4,19
	Tapped density	Dc	g/ml	0,542	0,544	0,541	0,542	5,42	5,44	5,41	5,42
**Compressibility**	Interparticle porosity	Ie	(-)	0,570	0,548	0,516	0,545	4,75	4,57	4,30	4,54
	Carr Index	IC	%	23,616	22,978	21,811	22,802	4,72	4,60	4,36	4,56
	Cohesion index	Icd	N ·	41,500	35,000	42,600	39,700	2,08	1,75	2,13	1,99
**Flowability**	Hausner Ratio	IH	(-)	1,309	1,298	1,279	1,295	8,45	8,51	8,61	8,52
	Angle of repose	(α)	º	42,345	43,876	40,077	42,099	1,53	1,22	1,98	1,58
	Flowability	t''	s	∞	∞	∞	∞	0,00	0,00	0,00	0,00
**Lubrication/stability**	Loss on drying	%HR	%	0,423	0,206	0,220	0,283	9,58	9,79	9,78	9,72
	Higroscopicity	%H	%	0,018	0,020	0,000	0,013	9,99	9,99	10,00	9,99
**Dosage/ lubrication**	Particles < 50 µ	%Pf	µ	13,002	11,260	12,320	12,194	7,40	7,75	7,54	7,56
	Homogenity Index	(Iθ)	-	0,009	0,009	0,008	0,009	4,50	4,40	4,15	4,35
							Parametric index (IP)	0,42	0,42	0,42	0,42
							Parametric profile (IPP)	5,21	5,18	5,21	5,20
							Good compression index (GCI)	4,96	4,94	4,96	4,95

Table S11. Parameters, radius values, and incidence factors for Linezolid Glenmarck batch GL-5. Individual experimental values (v1,v2 and v3) and mean of experimental values (v), radius of different parameters after applying factor described in Table 1 (R1, R2 and R3) and mean value of radius (R). Parameter index (IP), Parameter profile index (IPP) and Good compression index (GCI).

BATCH GL-5											
**Incidence**	**Parameter**	**Symbol**	**Unit**	**v1**	**v2**	**v3**	**v**	**R1**	**R2**	**R3**	**R**
**Dimensions**	Bulk density	Da	g/ml	0,424	0,416	0,418	0,419	4,24	4,16	4,18	4,19
	Tapped density	Dc	g/ml	0,558	0,544	0,546	0,549	5,58	5,44	5,46	5,49
**Compressibility**	Interparticle porosity	Ie	(-)	0,566	0,566	0,561	0,564	4,72	4,71	4,67	4,70
	Carr Index	IC	%	24,014	23,529	23,443	23,662	4,80	4,71	4,69	4,73
	Cohesion index	Icd	N ·	40,100	40,500	36,000	38,867	2,01	2,03	1,80	1,94
**Flowability**	Hausner Ratio	IH	(-)	1,316	1,308	1,306	1,310	8,42	8,46	8,47	8,45
	Angle of repose	(α)	º	42,769	42,202	44,940	43,304	1,45	1,56	1,01	1,34
	Flowability	t''	s	∞	∞	∞	∞	0,00	0,00	0,00	0,00
**Lubrication/stability**	Loss on drying	%HR	%	0,344	0,229	0,328	0,300	9,66	9,77	9,67	9,70
	Higroscopicity	%H	%	0,015	0,010	0,010	0,012	9,99	10,00	10,00	9,99
**Dosage/ lubrication**	Particles < 50 μ	%Pf	μ	12,366	11,441	12,240	12,016	7,53	7,71	7,55	7,60
	Homogenity Index	(Iθ)	-	0,008	0,008	0,009	0,008	4,15	3,95	4,50	4,20
				Parametric index (IP)				0,42	0,42	0,42	0,42
				Parametric profile (IPP)				5,21	5,21	5,17	5,20
				Good compression index (GCI)				4,96	4,96	4,92	4,95

Table S12. Parameters, radius values, and incidence factors for Linezolid Glenmarck batch GL-6. Individual experimental values (v1,v2 and v3) and mean of experimental values (v), radius of different parameters after applying factor described in Table 1 (R1, R2 and R3) and mean value of radius (R). Parameter index (IP), Parameter profile index (IPP) and Good compression index (GCI).

**BATCH GL-6**											
**Incidence**	**Parameter**	**Symbol**	**Unit**	**v1**	**v2**	**v3**	**v**	**R1**	**R2**	**R3**	**R**
**Dimensions**	Bulk density	Da	g/ml	0,420	0,423	0,425	0,423	4,20	4,23	4,25	4,23
	Tapped density	Dc	g/ml	0,556	0,544	0,557	0,552	5,56	5,44	5,57	5,52
**Compressibility**	Interparticle porosity	Ie	(-)	0,582	0,525	0,558	0,570	4,85	4,38	4,65	4,63
	Carr Index	IC	%	24,460	22,214	23,698	23,458	4,89	4,44	4,74	4,69
	Cohesion index	Icd	N ·	42,200	39,200	35,200	38,867	2,11	1,96	1,76	1,94
**Flowability**	Hausner Ratio	IH	(-)	1,324	1,286	1,311	1,307	8,38	8,57	8,45	8,47
	Angle of repose	(α)	º	43,369	39,035	40,821	41,075	1,33	2,19	1,84	1,78
	Flowability	t''	s	∞	∞	∞	∞	0,00	0,00	0,00	0,00
**Lubrication/stability**	Loss on drying	%HR	%	0,355	0,299	0,250	0,301	9,65	9,70	9,75	9,70
	Higroscopicity	%H	%	0,015	0,010	0,030	0,018	9,99	10,00	9,99	9,99
**Dosage/ lubrication**	Particles < 50 µ	%Pf	µ	12,578	10,683	10,688	11,316	7,48	7,86	7,86	7,74
	Homogenity Index	(Iθ)	-	0,008	0,008	0,007	0,008	4,10	4,15	3,65	3,97
				Parametric index (IP)				0,42	0,42	0,42	0,42
				Parametric profile (IPP)				5,21	5,24	5,21	5,22
				Good compression index (GCI)				4,96	4,99	4,96	4,97

Table S13. Parameters, radius values, and incidence factors for Linezolid USV batch US-4. Individual experimental values (v1,v2 and v3) and mean of experimental values (v), radius of different parameters after applying factor described in Table 1 (R1, R2 and R3) and mean value of radius (R). Parameter index (IP), Parameter profile index (IPP) and Good compression index (GCI).

BATCH US-4											
**Incidence**	**Parameter**	**Symbol**	**Unit**	**v1**	**v2**	**v3**	**v**	**R1**	**R2**	**R3**	**R**
**Dimensions**	Bulk density	Da	g/ml	0,258	0,262	0,255	0,258	2,58	2,62	2,55	2,58
	Tapped density	Dc	g/ml	0,356	0,357	0,365	0,359	3,56	3,57	3,65	3,59
**Compressibility**	Interparticle porosity	Ie	(-)	1,067	1,016	1,182	1,088	8,89	8,46	9,85	9,07
	Carr Index	IC	%	27,528	26,611	30,137	28,092	5,51	5,32	6,03	5,62
	Cohesion index	Icd	N ·	0,000	0,000	0,000	0,000	0,00	0,00	0,00	0,00
**Flowability**	Hausner Ratio	IH	(-)	1,380	1,363	1,431	1,391	8,10	8,19	7,84	8,04
	Angle of repose	(α)	º	46,418	44,051	45,201	45,223	0,72	1,19	0,96	0,96
	Flowability	t''	s	∞	∞	∞	∞	0,00	0,00	0,00	0,00
**Lubrication/stability**	Loss on drying	%HR	%	0,407	0,321	0,452	0,393	9,59	9,68	9,55	9,61
	Higroscopicity	%H	%	0,014	0,020	0,024	0,019	9,99	9,99	9,99	9,99
**Dosage/ lubrication**	Particles < 50 µ	%Pf	µ	2,998	3,602	3,069	3,223	9,40	9,28	9,39	9,36
	Homogenity Index	(Iθ)	--	0,020	0,017	0,019	0,019	9,95	8,40	9,40	9,25
							Parametric index (IP)	0,58	0,58	0,58	0,58
							Parametric profile (IPP)	5,69	5,56	5,77	5,67
							Good compression index (GCI)	5,42	5,29	5,49	5,40

Table S14. Parameters, radius values, and incidence factors for Linezolid USV batch US-5. Individual experimental values (v1,v2 and v3) and mean of experimental values (v), radius of different parameters after applying factor described in Table 1 (R1, R2 and R3) and mean value of radius (R). Parameter index (IP), Parameter profile index (IPP) and Good compression index (GCI).

BATCH US-5											
**Incidence**	**Parameter**	**Symbol**	**Unit**	**v1**	**v2**	**v3**	**v**	**R1**	**R2**	**R3**	**R**
**Dimensions**	Bulk density	Da	g/ml	0,262	0,249	0,253	0,255	2,62	2,49	2,53	2,55
	Tapped density	Dc	g/ml	0,356	0,341	0,357	0,351	3,56	3,41	3,57	3,51
**Compressibility**	Interparticle porosity	Ie	(-)	1,008	1,084	1,151	1,081	10,00	9,03	9,60	9,54
	Carr Index	IC	%	26,404	26,979	29,132	27,505	5,28	5,40	5,83	5,50
	Cohesion index	Icd	N •	0	0	0	0,000	0,00	0,00	0,00	0,00
**Flowability**	Hausner Ratio	IH	(-)	1,359	1,369	1,411	1,380	8,21	8,15	7,94	8,10
	Angle of repose	(α)	º	46,476	43,122	45,312	44,970	0,70	1,38	0,94	1,01
	Flowability	t''	s	∞	∞	∞	∞	0,00	0,00	0,00	0,00
**Lubrication/stability**	Loss on drying	%HR	%	0,311	0,378	0,392	0,360	9,69	9,62	9,61	9,64
	Higroscopicity	%H	%	0,09	0	0,03	0,040	9,96	10,00	9,99	9,98
**Dosage/ lubrication**	Particles < 50 μ	%Pf	μ	2,054	3,124	2,719	2,632	9,59	9,38	9,46	9,47
	Homogenity Index	(Iθ)	-	0,0192	0,0182	0,0178	0,018	9,60	9,10	8,90	9,20
					Parametric index (IP)			0,58	0,58	0,58	0,58
					Parametric profile (IPP)			5,77	5,66	5,70	5,71
					Good compression index (GCI)			5,49	5,39	5,42	5,43

Table S15. Parameters, radius values, and incidence factors for Linezolid USV batch US-6. Individual experimental values (v1,v2 and v3) and mean of experimental values (v), radius of different parameters after applying factor described in Table 1 (R1, R2 and R3) and mean value of radius (R). Parameter index (IP), Parameter profile index (IPP) and Good compression index (GCI).

BATCH US-6											
**Incidence**	**Parameter**	**Symbol**	**Unit**	**v1**	**v2**	**v3**	**v**	**R1**	**R2**	**R3**	**R**
**Dimensions**	Bulk density	Da	g/ml	0,255	0,247	0,258	0,253	2,55	2,47	2,58	2,53
	Tapped density	Dc	g/ml	0,359	0,347	0,362	0,356	3,59	3,47	3,62	3,56
**Compressibility**	Interparticle porosity	Ie	(-)	1,136	1,167	1,114	1,139	10,00	9,72	9,28	9,67
	Carr Index	IC	%	28,969	28,818	28,729	28,839	5,79	5,76	5,75	5,77
	Cohesion index	Icd	N •	0	0	0	0,000	0,00	0,00	0,00	0,00
**Flowability**	Hausner Ratio	IH	(-)	1,408	1,405	1,403	1,405	7,96	7,98	7,98	7,97
	Angle of repose	(α)	º	46,186	44,507	45,204	45,299	0,76	1,10	0,96	0,94
	Flowability	t''	s	∞	∞	∞	∞	0,00	0,00	0,00	0,00
**Lubrication/stability**	Loss on drying	%HR	%	0,306	0,358	0,413	0,359	9,69	9,64	9,59	9,64
	Higroscopicity	%H	%	0,09	0,02	0,03	0,047	9,96	9,99	9,99	9,98
**Dosage/ lubrication**	Particles < 50 µ	%Pf	µ	2,59	2,778	2,719	2,696	9,48	9,44	9,46	9,46
	Homogenity Index	($I\theta$)	-	0,0182	0,0196	0,0178	0,019	9,10	9,80	8,90	9,27
					Parametric index (IP)			0,58	0,58	0,58	0,58
					Parametric profile (IPP)			5,74	5,78	5,67	5,73
					Good compression index (GCI)			5,47	5,50	5,40	5,46

Table S16. Parameters, radius values, and incidence factors for Linezolid UQUIFA batch UQ-4. Individual experimental values (v1,v2 and v3) and mean of experimental values (v), radius of different parameters after applying factor described in Table 1 (R1, R2 and R3) and mean value of radius (R). Parameter index (IP), Parameter profile index (IPP) and Good compression index (GCI).

BATCH UQ-4											
**Incidence**	**Parameter**	**Symbol**	**Unit**	**v1**	**v2**	**v3**	**v**	**R1**	**R2**	**R3**	**R**
**Dimensions**	Bulk density	Da	g/ml	0,228	0,227	0,232	0,229	2,28	2,27	2,32	2,29
	Tapped density	Dc	g/ml	0,258	0,26	0,268	0,262	2,58	2,60	2,68	2,62
**Compressibility**	Interparticle porosity	Ie	(-)	0,510	0,559	0,579	0,549	4,25	4,66	4,83	4,58
	Carr Index	IC	%	11,628	12,692	13,433	12,584	2,33	2,54	2,69	2,52
	Cohesion index	Icd	N •	0	0	0	0,000	0,00	0,00	0,00	0,00
**Flowability**	Hausner Ratio	IH	(-)	1,132	1,145	1,155	1,144	9,34	9,27	9,22	9,28
	Angle of repose	(α)	º	45,24	46,17	43,25	44,887	0,95	0,77	1,35	1,02
	Flowability	t''	s	∞	∞	∞	∞	0,00	0,00	0,00	0,00
**Lubrication/stability**	Loss on drying	%HR	%	0,31	0,166	0,532	0,336	9,69	9,83	9,47	9,66
	Higroscopicity	%H	%	0,057	0,05	0,049	0,052	9,97	9,98	9,98	9,97
**Dosage/ lubrication**	Particles < 50 μ	%Pf	μ	34,134	31,13	32,353	32,539	3,17	3,77	3,53	3,49
	Homogenity Index	(Iθ)	-	0,0053	0,0043	0,0048	0,005	2,62	2,15	2,40	2,39
							Parametric index (IP)	0,25	0,25	0,25	0,25
							Parametric profile (IPP)	3,93	3,99	4,04	3,99
							Good compression index (GCI)	3,74	3,80	3,84	3,79

Table S17. Parameters, radius values, and incidence factors for Linezolid UQUIFA batch UQ-5. Individual experimental values (v1,v2 and v3) and mean of experimental values (v), radius of different parameters after applying factor described in Table 1 (R1, R2 and R3) and mean value of radius (R). Parameter index (IP), Parameter profile index (IPP) and Good compression index (GCI).

BATCH UQ-5											
**Incidence**	**Parameter**	**Symbol**	**Unit**	**v1**	**v2**	**v3**	**v**	**R1**	**R2**	**R3**	**R**
**Dimensions**	Bulk density	Da	g/ml	0,227	0,229	0,235	0,230	2,27	2,29	2,35	2,30
	Tapped density	Dc	g/ml	0,258	0,269	0,263	0,263	2,58	2,69	2,63	2,63
**Compressibility**	Interparticle porosity	Ie	(-)	0,529	0,649	0,453	0,544	4,41	5,41	3,78	4,53
	Carr Index	IC	%	12,016	14,870	10,646	12,511	2,40	2,97	2,13	2,50
	Cohesion index	Icd	N •	0	0	0	0,000	0,00	0,00	0,00	0,00
**Flowability**	Hausner Ratio	IH	(-)	1,137	1,175	1,119	1,143	9,32	9,13	9,40	9,28
	Angle of repose	(α)	º	42,432	46,774	44,53182	44,579	1,51	0,65	1,09	1,08
	Flowability	t''	s	∞	∞	∞	∞	0,00	0,00	0,00	0,00
**Lubrication/stability**	Loss on drying	%HR	%	0,744	0,673	0,076	0,498	9,26	9,33	9,92	9,50
	Higroscopicity	%H	%	0,06	0,048	0,028	0,045	9,97	9,98	9,99	9,98
**Dosage/ lubrication**	Particles < 50 μ	%Pf	μ	33,46	33,232	32,441	33,044	3,31	3,35	3,51	3,39
	Homogenity Index	$(I\theta)$		0,0043	0,0057	0,005	0,005	2,17	2,85	2,50	2,51
							Parametric index (IP)	0,25	0,25	0,25	0,25
							Parametric profile (IPP)	3,93	4,05	3,94	3,98
							Good compression index (GCI)	3,74	3,86	3,75	3,79

Table S18. Parameters, radius values, and incidence factors for Linezolid UQUIFA batch UQ-6. Individual experimental values (v1,v2 and v3) and mean of experimental values (v), radius of different parameters after applying factor described in Table 1 (R1, R2 and R3) and mean value of radius (R). Parameter index (IP), Parameter profile index (IPP) and Good compression index (GCI).

BATCH UQ-6											
**Incidence**	**Parameter**	**Symbol**	**Unit**	**v1**	**v2**	**v3**	**v**	**R1**	**R2**	**R3**	**R**
**Dimensions**	Bulk density	Da	g/ml	0,226	0,237	0,235	0,233	2,26	2,37	2,35	2,33
	Tapped density	Dc	g/ml	0,257	0,268	0,267	0,264	2,57	2,68	2,67	2,64
**Compressibility**	Interparticle porosity	Ie	(-)	0,534	0,488	0,510	0,511	4,45	4,07	4,25	4,25
	Carr Index	IC	%	12,062	11,567	11,985	11,871	2,41	2,31	2,40	2,37
	Cohesion index	Icd	N •	0	0	0	0,000	0,00	0,00	0,00	0,00
**Flowability/**	Hausner Ratio	IH	(-)	1,137	1,131	1,136	1,135	9,31	9,35	9,32	9,33
	Angle of repose	(α)	º	44,758	45,097	43,271	44,375	1,05	0,98	1,35	1,12
	Flowability	t''	s	∞	∞	∞	∞	0,00	0,00	0,00	0,00
**Lubrication/stability**	Loss on drying	%HR	%	0,522	0,234	0,349	0,368	9,48	9,77	9,65	9,63
	Higroscopicity	%H	%	0,016	0,029	0,048	0,031	9,99	9,99	9,98	9,98
**Dosage/ lubrication**	Particles < 50 μ	%Pf	μ	34,714	31,51	32,171	32,798	3,06	3,70	3,57	3,44
	Homogenity Index	(Iθ)	-	0,0037	0,0048	0,0052	0,005	1,85	2,40	2,60	2,28
							Parametric index (IP)	0,25	0,25	0,25	0,25
							Parametric profile (IPP)	3,87	3,97	4,01	3,95
							Good compression index (GCI)	3,68	3,78	3,82	3,76

Table S19. Composition of the Linezolid tablets produced with direct compression technology, API batch: US-3.

Components	Percentage (%)	Quantity (mg)/tablet
Linezolid	65,9%	600
Microcel ® MC	19,8%	180
Emcompress ®	11,0%	100
Talc	2,2%	20
Magnesium stearate	1,0%	8,8
Colloidal silicon dioxide	0,1%	1,2
**Total weight tablet (mg)**	**100%**	**910**

Table S20. Critical quality attributes of Linezolid tablets (Batch US 3.3), USV source obtained by direct compression.

Components	Percentage (%)	Quantity (mg)/tablet
Linezolid	70,6%	600
Microcel ® MC	16,5%	140
Emcompress ®	9,4%	80
Talc	2,4%	20
Magnesium stearate	1,0%	8,8
Colloidal silicon dioxide	0,1%	1,2
**Total weight tablet (mg)**	**100%**	**850**
